# Exploration of Extracellular Vesicle miRNAs, Targeted mRNAs and Pathways in Prostate Cancer: Relation to Disease Status and Progression

**DOI:** 10.3390/cancers14030532

**Published:** 2022-01-21

**Authors:** Maija Puhka, Lisse Thierens, Daniel Nicorici, Tarja Forsman, Tuomas Mirtti, Taija af Hällström, Elina Serkkola, Antti Rannikko

**Affiliations:** 1 HiPrep and EV Core, Institute for Molecular Medicine Finland FIMM, University of Helsinki, 00290 Helsinki, Finland; Lisse.Thierens@UGent.be; 2 Orion Pharma, Orion Corporation, 02200 Espoo, Finland; Daniel.Nicorici@orionpharma.com (D.N.); Tarja.Forsman@orionpharma.com (T.F.); Elina.Serkkola@orionpharma.com (E.S.); 3 Department of Pathology, HUS Diagnostic Centre, Helsinki University Hospital, 00290 Helsinki, Finland; Tuomas.Mirtti@hus.fi; 4 Research Program in Systems Oncology, Faculty of Medicine, University of Helsinki, 00290 Helsinki, Finland; 5 AstraZeneca, 02600 Espoo, Finland; Taija.afhallstrom@astrazeneca.com; 6 Department of Urology, University of Helsinki and Helsinki University Hospital, 00290 Helsinki, Finland

**Keywords:** prostate cancer, extracellular vesicles, exosomes, micro RNA, messenger RNA, sequencing, progression, pathway

## Abstract

**Simple Summary:**

Prostate cancer lacks non-invasive specific biomarkers for aggressive disease. Urinary extracellular vesicles (uEV) could provide such markers; however, due to technical challenges, little is known regarding the pathogenesis pathways reflected in uEV. We performed a miRNA, target mRNA and pathway study focused on uEV, exploring the differences between cancer (1) status groups (Gleason score) and (2) progression groups. The uEV provided a surprisingly comprehensive presentation of differentially expressed miRNAs, target mRNAs and pathogenesis pathways. The miRNAs associated with prostate cancer status or progression were mostly unique, but still targeted overlapping sets of signalling, resistance, hormonal and immune pathways. Interestingly, mRNA targets of the key miRNAs (miR-892a, miR-223-3p, miR-146a-5p) were widely expressed in both uEV and plasma EV from PCa patients. The study thus suggests that uEV carry a vast presentation of PCa status and progression-linked RNAs that are worth further exploration in large personalized medicine trials.

**Abstract:**

Background: Prostate cancer (PCa) lacks non-invasive specific biomarkers for aggressive disease. We studied the potential of urinary extracellular vesicles (uEV) as a liquid PCa biopsy by focusing on the micro RNA (miRNA) cargo, target messenger RNA (mRNA) and pathway analysis. Methods: We subjected uEV samples from 31 PCa patients (pre-prostatectomy) to miRNA sequencing and matched uEV and plasma EV (pEV) from three PCa patients to mRNA sequencing. EV quality control was performed by electron microscopy, Western blotting and particle and RNA analysis. We compared miRNA expression based on PCa status (Gleason Score) and progression (post-prostatectomy follow-up) and confirmed selected miRNAs by quantitative PCR. Expression of target mRNAs was mapped in matched EV. Results: Quality control showed typical small uEV, pEV, RNA and EV-protein marker enriched samples. Comparisons between PCa groups revealed mostly unique differentially expressed miRNAs. However, they targeted comprehensive and largely overlapping sets of cancer and progression-associated signalling, resistance, hormonal and immune pathways. Quantitative PCR confirmed changes in miR-892a (Gleason Score 7 vs. ≥8), miR-223-3p (progression vs. no progression) and miR-146a-5p (both comparisons). Their target mRNAs were expressed widely in PCa EV. Conclusions: PCa status and progression-linked RNAs in uEV are worth exploration in large personalized medicine trials.

## 1. Introduction

Prostate cancer (PCa), the most common malignancy of men in Finland and other Western countries, is one of the leading causes of cancer-related deaths. For PCa diagnosis, tumour tissue biopsies are the gold standard. However, biopsies cause problems due to their invasiveness, low yields of tissue, e.g., from bone metastasis, or insufficient representation across the heterogeneous tumour foci [1]. In contrast, the widely applied PCa biomarker, prostate-specific antigen (PSA), can easily be measured from serum for clinical diagnostics, prognostics and disease or therapy monitoring. The specificity of PSA is unfortunately poor, leading to over-diagnosis or -treatment [2,3]. Thus, in need of better PCa biomarkers, the PCa research field has turned its focus to liquid biopsies.

Urinary extracellular vesicles (uEV) are membrane-enclosed vesicles derived mainly from the cells of the genitourinary system. They carry internal and external molecular cargo such as nucleic acids, proteins, lipids and metabolites [4,5]. UEV are of interest, e.g., due to their potential functions in intercellular communication and cargo, which is considered as a non-invasive source of biomarkers for different pathological conditions, including PCa [6,7]. Particularly, the microRNA (miRNA) content of uEV from patients with PCa has raised considerable interest [8]. Most studies have evaluated the potential of uEV miRNAs in PCa detection (diagnosis), and included uEV technology development [9,10,11,12]. However, less is known about how PCa progression or therapy resistance could change the contents of the uEV in clinical samples. Few studies indicate changes in urine or uEV miRNAs associated with biochemical recurrence [13,14] or between low vs. high grade and localized vs. metastatic cancers [15,16,17]. A recent study elucidated the effect of androgen manipulation (dihydrotestosterone and enzalutamide) on the EV contents from LnCaP cells, including miRNAs [18]. However, the discovered miRNA biomarker candidates have differed between studies, rendering it difficult to evaluate uEV’s potential to provide information regarding the PCa pathogenesis pathways. Equally little is known regarding whether analysis of miRNA with their target mRNA from EV adds valuable insights into PCa pathogenesis, or how EV from urine and plasma differ in their RNA cargo. These are interesting questions because, along with their own challenges, both sample types also have unique potential in PCa research and diagnostics, e.g., urine as a source of EV from primary tumour and blood as a source of EV from distant metastasis [19].

Technically, RNA research into uEV is challenging. The best practices in EV pipelines are currently still developing, and they include pre-analytics (sample collection, processing and storage), EV isolation, RNA detection and data normalization/analysis [5,7,20,21]. The International Society of Extracellular Vesicles (ISEV) has launched several standardization efforts to expedite the development, including, e.g., Minimal Information for Studies of Extracellular Vesicles (MISEV) guidelines, and targeted ISEV position papers [5,22]. However, so far, the heterogeneity of the uEV pipelines, on top of the heterogeneity of the PCa tumours and study designs, likely explains why the discovered miRNA biomarker candidates differ [8]. For example, heterogeneity of the isolated uEV and copurified non-EV materials from different isolation workflows impact the miRNA results [7,23,24]. As the average sizes, biogenesis routes and cargo of small and large EV differ [22,25,26,27], targeting small EV for biomarker analysis is one strategy to limit heterogeneity of the EV and dissect exosomal messages. Prior studies have further shown that PCa cells secrete large quantities of exosome-sized EV, e.g., due to hypoxia and low pH, typical for the solid tumour microenvironment [28,29]. Finally, targeting small EV also allows better sample purification i.e., preclearing steps to remove cells, bacteria or other bigger contaminants before EV purification.

In this study, we focused on small uEV enriched samples to explore whether miRNAs and their target mRNAs could provide biomarker candidates and information regarding the different pathogenesis pathways linked to PCa status or progression.

## 2. Materials and Methods

### 2.1. Study Participants and Groups

Urine or EDTA plasma samples and/or clinical data were obtained from consented donors of the Helsinki Biobank, Helsinki Urological Biobank, and the SalWe “Get It Done” research project and from the FinnProstata IX study. 

For the main study, PCa patients (*n* = 30, age range 51–75) were classified into three cancer status groups according to the following criteria: Group A had a Gleason score of ≥8; Group B had a Gleason score of 7 and were lymph node metastasis positive and/or had margin positive prostatectomy tissue sample and/or PSA > 0.2 ng/mL after radical prostatectomy (post-RP); Group C patients had a Gleason score of 7, were lymph node and margin negative and their PSA remained < 0.2 ng/mL post-RP. PSA measurements were taken ≥33 days post-RP. Healthy technical/biological controls; Group D (*n* = 10) were young (<45 years) asymptomatic men.

The outcomes of the patients were followed for 4–7 years post-RP. Then, PCa patients were divided into new groups according to disease progression. Group I contained individuals who died due to PCa and/or had metastasis and/or were hormonally treated, Group II obtained secondary treatment and/or had a biochemical recurrence and Group III had no progression during follow-up.

For the correlation study, the PCa patients (*n* = 3) were classified according to the above status criteria with the addition of group E having metastatic castration resistant PCa at the time of urine and plasma sample collection. The Gleason scores of Group E were determined through needle biopsies as the patients did not undergo RP.

### 2.2. Urine and Plasma Sample Collections and EV Isolation

For the main study, all urine samples were from spot mid-stream urine collections obtained before RP (pre-RP, 0–43 days before, urine collection dates available for 25 out of 30 PCa patients). Samples were kept cold (ice, cold package or +4 °C) during storage and delivery to the laboratory (average 4–6 h), where they were centrifuged at 1800× *g*, 10 min, +4 °C. Supernatants were frozen at −80 °C or liquid nitrogen vapor phase. Samples were isolated using a modification of protocol described producing small uEV enriched samples [4,7,30]. Briefly, urine samples were vortexed for 90 s and centrifuged at 8000× *g* at +4 °C for 15 min using a fixed angle AG-6512C rotor (Kubota Corp., Tokyo, Japan). Supernatants were filtered with Whatman 1.2 μm cellulose acetate syringe filters (GE Healthcare, Buckinghamshire, UK) and 18 mL of processed urine diluted to 30 mL with PBS, which was then centrifuged at 4 °C for 90 min at 100,000× *g* (maximum breaking) in a SW28 or SW32 rotor, k-factor 251 and 266, respectively (Beckman Coulter, Inc., Brea, CA, USA). Supernatants were discarded and the pellets suspended in filtered PBS (0.22 μm PES filter; Jet Bio-Filtration, Guangzhou, China) and stored in protein or DNA LowBind tubes (Eppendorf) at −80 °C. For uEV quality control with nanoparticle tracking analysis (NTA), Western blotting, electron microscopy (EM) or sequencing, 9–18 mL of urine available for six patient samples, or 18 mL of pooled healthy control samples (HcI, *n* = 11 men, and HcII, *n* = 9, 2 men and 7 women) were subjected to uEV isolation following the same protocol.

For the correlation study, matched urine and EDTA plasma samples were collected from all non-fasting donors on the same day. P33 gave samples on the day of RP (pre-RP) and 6 months post-RP, and P34 and P35 gave samples 2–3 weeks after and before needle biopsy, respectively. Urine was collected, processed and stored similarly as above and uEV isolated from 17–30 mL urine as described for small uEV enriched samples [4,7,30]. Blood was drawn in K2F EDTA BD Vacutainer^®^ tubes (BD Biosciences, Franklin Lakes, NJ, USA) and centrifuged at 2000× *g* for 10 min, and plasma aliquots were frozen at −80 °C or the liquid nitrogen vapor phase. ExoEasy Maxi kit (Qiagen, Hilden, Germany) was used for EV isolation from 0.9–1.5 mL of plasma, according to the manufacturer’s instructions, including the pre-filtration (0.8 µm pore size, Millex AA, Merck Millipore, Burlington, MA, USA) of plasma. Eluted EV were frozen at −80 °C in DNA LowBind tubes (Eppendorf, Hamburg, Germany). All samples of HC11 were processed as three technical replicates. For uEV quality control with EM, Western blotting, or sequencing, plasma from a healthy man, HC12, pooled healthy donor urine samples (HcIII, *n* = 3 men) and matched plasma and urine samples from a healthy woman, HC13, were collected and processed similarly as correlation study samples.

### 2.3. EV Characterization by EM, Western Blotting and NTA

Negative staining for transmission EM was performed as previously described [4,30]. Briefly, uEV or pEV were loaded on Formvar/Carbon 200 mesh TH, Copper grids (Ted Pella Inc., Redding, CA, USA), fixed with 2% paraformaldehyde (Electron Microscopy Sciences, Hatfield, PA, USA) and stained with 2% neutral uranyl acetate and embedded in methyl cellulose uranyl acetate mixture (1.8/0.4%). Images were captured with a Gatan Orius SC 1000B CCD-camera (Gatan Inc., Pleasanton, CA, USA) in Jeol JEM-1400 (Jeol Ltd., Tokyo, Japan) operating at 80 kV using an image size of 4008 × 2672 pixels.

For Western blotting, uEV derived from equal volumes of urine, 33 µL of pEV or 20 µg of protein from LnCaP cells were denatured in Laemmli buffer and loaded onto 4–20% Mini-PROTEAN^®^ TGX Stain-Free Protein Gels (Bio-Rad laboratories Inc., Hercules, CA, USA) together with BlueStar PLUS Prestained Protein Marker (Nippon Genetics Co., Tokyo, Japan). Protein quantification, SDS-PAGE and Western blotting for CD9, CD59, CD63, TSG101, Podocalyxin (PDX), calnexin and Tamm-Horsfall protein (THP) were performed as previously described [4,7,30].

The particle concentration and size distribution were analysed from uEV preparations by an NTA instrument LM14C equipped with a violet (405 nm, 70 mW) laser (Malvern Instruments Ltd., Malvern, UK) and an sCMOS camera (Hamamatsu Photonics K.K., Hamamatsu, Japan). Capture settings were temperature 18.2–19.9 °C, viscosity 1.035–1.043 cP and camera level 14. EV samples were diluted with filtered PBS, and five 30s videos were recorded with 36–102 particles/frame. Analysis was carried out with auto settings and detection threshold 4 in Nanosight software 3.1 (Malvern Instruments Ltd., Malvern, UK).

### 2.4. RNA Extraction and miRNA Sequencing in the Main Study

Isolated uEV samples were submitted to Qiagen Genomic Services (Hilden, Germany) for miRNA sequencing (miRNAseq). Total uEV RNA was extracted using Qiagen’s miRNeasy serum/plasma kit according to the manufacturer’s instructions. The library preparation was carried out using the QIAseq miRNA Library Kit with unique molecular identifiers (UMI) tagging (Qiagen). The cDNA was amplified using PCR (22 cycles). Library quality was monitored using either Bioanalyzer 2100 (Agilent Technologies, Santa Clara, CA, USA) or TapeStation 4200 (Agilent). Sequencing was performed with NextSeq500 and 75 basepairs (bp) single-end reads (Illumina Inc., San Diego, CA, USA).

Raw data was de-multiplexed and FASTQ files for each sample were generated using the bcl2fastq software (Illumina Inc., San Diego, CA, USA). FASTQ data were checked using the FastQC tool. Sequence annotation was performed using Homo sapiens reference genome GRCh37 and miRBase 20 as an annotation reference. Bowtie2 (2.2.2) was used for mapping the reads. Quality was monitored through UMI correction, quality score (Q-score) of incorrect base call probability using 30 as a cut-off (an error probability < 0.001), read length (>15 nucleotides (nt) as cut-off) and mapping to reference genome or miRbase, as well as through read numbers (Table S1).

Differential expression analysis was performed using the CLC Genomic Workbench version 20.0.4 (Qiagen) and EdgeR statistical software package (Bioconductor, http://bioconductor.org/, (accessed on 12 October 2020)). For normalisation, counts per million (CPM) or the trimmed mean of the M-values method (TMM) based on log-fold and absolute gene-wise changes in expression levels between samples were used [31]. Principal component analysis (PCA) was performed with CLC Genomic Workbench or R using normalized quantifications. The Kyoto Encyclopaedia of Genes and Genomes (KEGG) pathways were analysed with miRWalk [32] using all nominal significantly differentially expressed (DE) miRNAs (FDR (false discovery rate) *p* or *p* < 0.05) as input and filtering for 3′UTR (untranslated region) interactions and for targets found using TargetScan, miRDB and miRTarBase. Tam 2.0 tool overrepresentation analysis [33,34] was also performed with all nominally significant DE miRNAs as inputs and the settings of all curated miRNAs as background, limited to a miRNA set size of two, including up- and down-regulated miRNA sets.

### 2.5. Quantitative PCR and Analysis

For technical confirmation of the miRNAseq results, the quantitative PCR (qPCR) of selected targets and reference candidate miRNAs were carried out at Qiagen (all tested assays listed in Table S2). The DE miRNAs were selected based on two or more of the following criteria: transcripts per million (TPM) ≥10 in all samples of a group, FDR *p* or *p* < 0.05, log2FC (fold change) >0.6 or <−0.6, as well as visual evaluation of variance and separation between groups. RNA (1.7 µL) was reverse transcribed using the miRCURY LNA RT Kit (Qiagen), and 50-fold dilutions of cDNA were assayed once for each miRNA on the miRCURY LNA miRNA PCR Custom panel using SYBR Green master mix. The amplification was performed in a LightCycler^®^ 480 Real-Time PCR System (Roche Diagnostics, Mannheim, Germany). The amplification curves were analysed using the Roche LC software. Quality control was performed at reverse transcription (RT) step by inclusion of RNA and DNA spike-ins, by melting curve and amplification efficiency analysis as well as by comparisons of Cq (quantification cycle) values to background level in the negative control samples (no template in the RT step). Inclusion criteria for analysis was that assays yielded signals ≥3 Cq lower than the negative control in at least 20% of samples, or <37 Cq in the case of there being no signal from negative control.

For normalization in assessing the miRNA expression, miR-103a-3p, -99b-5p, -151a-3p and let-7b-5p were selected as reference miRNAs based on NormFinder [35] stability values in the miRNAseq (Table S2) as well as through evidence of uEV expression and PCa literature survey (e.g., [7,11,20,36,37,38,39]) and non-differential expression between groups in first qPCR. Applied calculations were (1) Normalized delta Cq (dCq) = normalizer assays mean Cq − assay Cq, (2) Differences in expression levels = dCq(1) − dCq(2) = ddCq, and (3) Fold change = 2^ddCq. Receiver operator characteristic (ROC) curve analysis using the dCq values was conducted with EasyROC [40] using the default settings and Youden or ROC1 method for the cut-point analysis.

### 2.6. RNA Extraction and miRNA and mRNA Sequencing for the Corelation Study of Three Patients

Total RNA from uEV and pEV samples and 300 µL of plain plasma were isolated using a miRNEasy micro kit (Qiagen) according to the protocol for animal cells without DNAse treatment and with the exception that lysis was performed using Trizol LS (Life Technologies Corp., Carlsbad, CA, USA) due to its suitability for larger sample volumes. The quality and quantity of the RNA was monitored with a Bioanalyzer 2100 (Agilent) Pico kit.

The mRNA sequencing (mRNAseq) was performed as described [20]. Briefly, for sequencing libraries, 400 pg of total RNA were prepared with SMART-Seq v4 Ultra Low Input RNA Kit for Sequencing (Takara BIo Inc., Mountain View, CA, USA) and Nextera XT kit (Ilumina Inc., San Diego, CA, USA) according to the manufacturer’s protocols. Libraries were sequenced on the Illumina Hiseq 2000 platform (Illumina Inc.) as 100 bp cycle paired-end reads. The sequences of adaptors were removed from the reads and the reads were aligned on human genome (version GRCh38) using gene annotation from ensemble database version 81 using STAR [41]. The miRNA sequencing libraries were prepared using 1 ng of total RNA for SMARTer^®^ smRNA-Seq Kit for Illumina^®^ (Takara Bio USA, Inc., San Jose, CA, USA), and 15 pM loading concentration was then used in sequencing with MiSeq (Illumina Inc.) as 75 bp paired-end reads. The miRNAseq data analysis was carried out essentially as above but without UMI correction. The mRNA targets of miR-146a-5p, -223-3p and -892a were downloaded from miRWalk [32], including all targets found in TargetScan, miRDB and miRTarBase (accession date 22 December 2021). Expression level differences were calculated using CPM data from both miRNA- and mRNAseq (Tables S3 and S4).

### 2.7. Statistical Testing and Venn Analysis

Statistical testing for miRNAseq read numbers was carried out with Student’s t-test. The statistical significance of patient demographic and clinical data and DE miRNAs in miRNAseq, qPCR and pathway analyses were assessed using t-test or ANOVA with and without Benjamini–Hochberg FDR correction [42]. In overrepresentation analysis with Tam 2.0, significance was additionally tested with multiple correction via the Bonferroni method. In addition, in qPCR, the normality of the data was tested with the Shapiro–Wilk method [43]. In case of non-normally distributed data, a non-parametric unpaired Wilcoxon test [44] was used for assessing statistical significance; *p*-values < 0.05 were considered statistically significant. Venn analyses were carried out with a Venn diagram tool (https://www.vandepeerlab.org/?q=tools/venn-diagrams, (accessed on 30 December 2021)).

## 3. Results

### 3.1. Design of the Main Study

**Figure 1:**
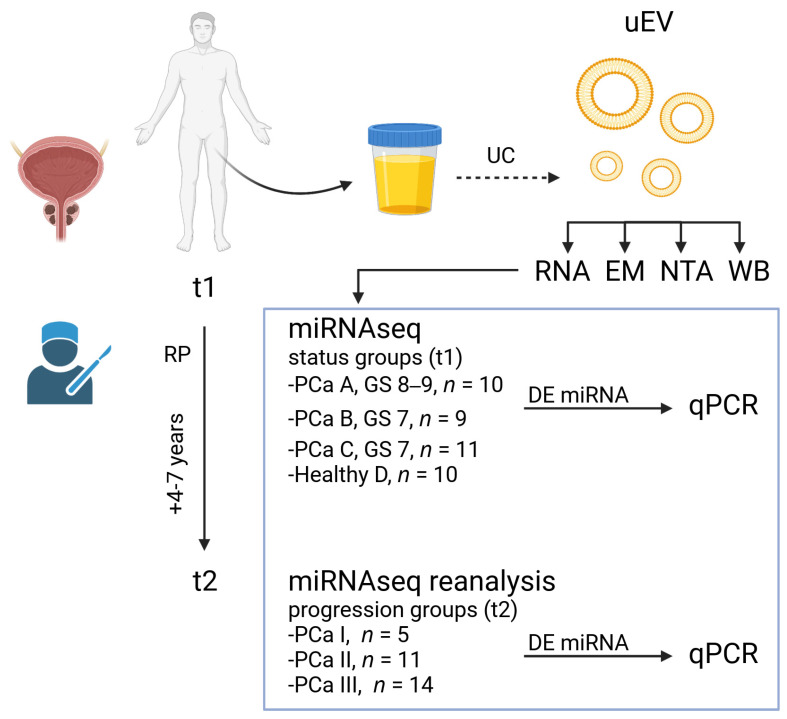
Outline of the main study. Study outline shows urine sample collection from prostate cancer (PCa) patients (time 1, t1) before radical prostatectomy (RP) and subsequent urinary extracellular vesicle (uEV) isolation with ultracentrifugation (UC) and miRNA sequencing (miRNAseq). The uEV quality control was carried out via RNA profiling, electron microscopy (EM), nanoparticle tracking analysis (NTA) and Western blotting (WB). After miRNAseq, differentially expressed (DE) miRNAs were compared between PCa status groups (A–C) and healthy technical controls (D). Groups A–C were based on Gleason scores (GS) 7–9, determined in prostatectomy tissue and other findings. Group B differed from C by more severe histological findings, metastasis or higher prostate specific antigen levels post-RP. Cancer progression was followed for 4–7 years post-RP and then (t2) patients were reclassified to groups (I–III) according to disease aggressiveness for reanalysis of the miRNAseq data. Groups I–III were based on death due to prostate cancer, metastasis or hormonal treatment (I), secondary treatment or biochemical recurrence (II) or no events (III) during follow-up time. Selected DE miRNAs from both analyses were confirmed by quantitative PCR (qPCR). Created with BioRender.com.

Our study aimed to clarify whether miRNA contents in uEV from PCa patients differed (1) between PCa status groups and (2) between PCa progression groups (Figure 1). For these aims, we prepared uEV and carried out uEV quality control and miRNAseq from the urine samples of 30 PCa patients prior to their radical prostatectomy (RP) and from 10 healthy non age-matched men as additional technical and biological controls. The Gleason score and other histological characteristics of cancer in the prostatectomy tissue, or PSA levels post-RP, were used for dividing the patients into cancer status groups (Table 1). Group A, with the most aggressive disease, had a Gleason score of ≥8 (*n* = 10). Group B (*n* = 9) had a Gleason score of 7 and possible or verified metastasis or elevated postoperative PSA. Group C (*n* = 11) had a Gleason score of 7 and no indication of metastasis or elevated postoperative PSA. The healthy group D consisted of younger men who had the minimal possibility of asymptomatic PCa. After prostatectomy, we followed the disease progression in the patients for 4–7 years and re-grouped the patients to form progression groups (Table 1). Patients in Group I (*n* = 5) had the most severe disease progression with death, metastasis and/or hormonal treatment, Group II (*n* = 11) obtained secondary treatment and/or had a biochemical recurrence and Group III (*n* = 14) had non-progressive disease. For both status and progression groups, we analysed DE miRNAs and their regulated pathways and confirmed the selected miRNAs by qPCR using the same set of samples.

### 3.2. uEV and miRNA Sequencing Quality

**Figure 2:**
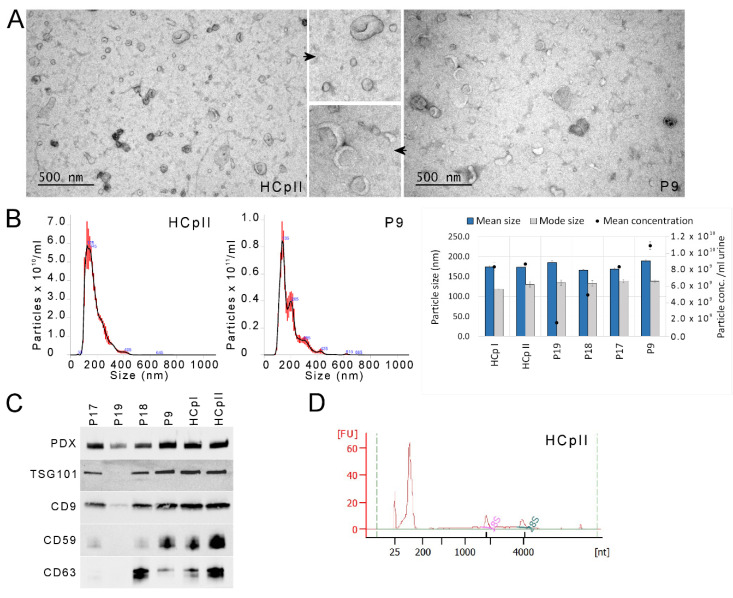
Quality control of uEV. (**A**) Representative electron micrographs (wide-field and close-up) and (**B**) representative nanoparticle tracking analysis histograms, as well as mean and mode size and mean concentration of particles in different study samples. (**C**) Same samples were subjected to analysis of uEV enriched protein markers by Western blotting. The uncropped Western blots have been shown in Figure S5. (**D**) Total RNA profile from HCpII uEV obtained by Bioanalyzer Pico assay. The status and progression groups of the patients were: P9—B and I, P17—C and II, P18—C and II, P19—C and III. Concentration (Conc.), healthy control pool I and II (HCpI and HCpII), patient (P), podocalyxin (PDX).

We isolated uEV using a modified ultracentrifugation (UC) protocol due to the low sampling volumes of the collected urine samples (18 mL as compared to 30 mL protocol used in, e.g., [7]. Thus, we performed a thorough quality control of the uEV from representative individual patient and pooled control samples by EM, NTA, Western blotting and RNA profiling analysis (Figure 2, *n* = 3–8 individual samples per method). The EM images showed similar small uEV enriched samples as before [4,7,20,30] with variable levels of THP, but no obvious other contaminants such as cellular remnants or intracellular organelles, including mitochondria. By NTA, particle mean (166–189 nm) and mode (118–139) sizes were similar between samples, while concentrations varied almost 10-fold (1.6 × 10^9^–1.1 × 10^10^ particles per ml urine), with both the minimum and maximum concentration detected in patient samples. Western blotting further confirmed the presence of uEV as the typical commonly enriched uEV markers; CD9, CD63, CD59, TSG-101 and PDX were detected in the samples, albeit in variable quantities. In addition, we detected the presence of THP and also some calnexin, an endoplasmic reticulum marker (Figure S1). Extracted total RNAs were analysed by BioAnalyzer 6000 Pico RNA assay and showed a small RNA peak between 25–300 nt and no or a small amount of 18S and 28S rRNA.

The RNAs were subjected to miRNAseq, which gave good quality sequences (Q-score > 30) and resulted in 17.4M average raw reads (range, 13.7–24.1 M) and 3.2 M average UMI corrected reads (range, 1.5–6.4 M, Figure S2). There were no statistically significant differences between read numbers from healthy controls and patients. The sequencing read distribution showed that an average of 30% (range, 7–57%) aligned to miRNAs, 8% (1–25%) to other small RNAs and 40% (17–66%) were either out- or unmapped. Under 1% aligned to predicted or putative miRNAs. The uEV from PCa patients yielded 2.2-, 2.7- and 8.3-fold more reads of miRNA, small RNA and predicted miRBase miRNA, respectively, than uEV from controls (*p* < 0.05). On average, we identified 461 (range, 341–545) miRNAs that were expressed at the ≥1 TPM level in all 40 samples. Out of these, 247 (range, 181–247) were expressed robustly at the ≥10 TPM level. Despite the larger miRNA read numbers from PCa uEV samples, the number of identified miRNAs did not differ significantly between PCa (average 467/251 miRNAs with 1/10 TPM, respectively) and healthy controls (average 445/238 miRNAs with 1/10 TPM, respectively). The two pools (HCpI and HCpII) used for quality control of the uEV isolation workflow (Figure 2) also produced good sequencing quality, with over 29 M raw reads, 7 M UMI corrected reads, 450/250 identified miRNAs (expressed at 1/10 TPM level) and a similar read distribution to the study samples (Figure S2D).

### 3.3. uEV from Prostate Cancer Patient Status Groups Differed in the Quantities of miRNAs Targeting Cancer and Progression-Linked Signaling, Resistance and Hormonal Pathways

We started miRNAseq data exploration of the PCa patient status groups (A–C) and healthy controls (D) by PCA focused on the 50 most variable miRNAs (Figure S3). PCA did not show clustering according to PCa, age or health status groups, but revealed some non-clustering PCa samples including an outlier, patient 30 (P30), in status group A. However, no clear status-based groupings were evident, even when P30 was removed from the analysis (Figure S3A,B). Hence, we decided to include this patient in the differential expression comparisons (Table S5), but also provide the data without P30 as additional supplementary information (Table S6).

**Figure 3:**
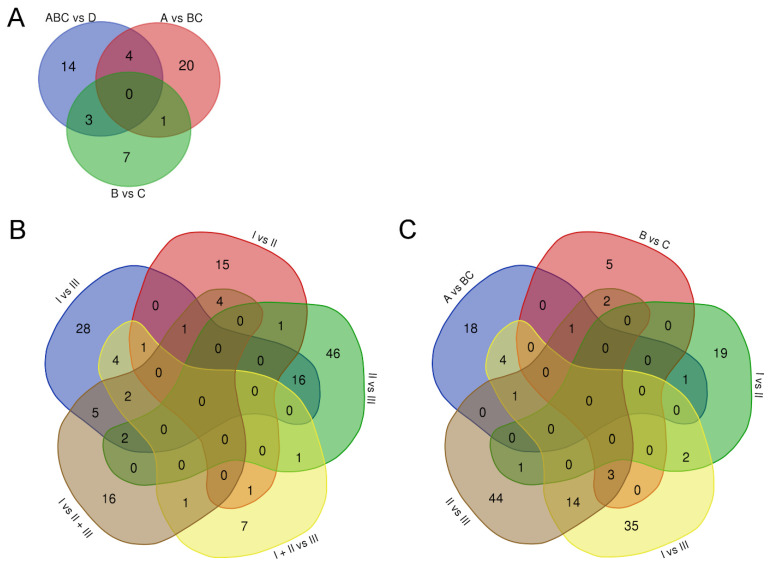
Venn analysis of differentially expressed miRNAs from miRNAseq. Comparison of changed miRNAs between (**A**) prostate cancer status groups (A–C) or healthy group (D) and (**B**) prostate cancer progression groups (I–III) and (**C**) both status and progressions groups.

DE analysis found 25 differential miRNAs between PCa patients with a Gleason score of ≥8 vs. those with a score of 7 (A vs. BC combined) (FDR *p* or *p* < 0.05, Table S5). The two Gleason score 7 status groups, B and C, differed by only 11 miRNAs (FDR *p* or *p* < 0.05, Table S5). We additionally checked the most DE miRNAs, 21, between all PCa patients (ABC combined, *n* = 30) and the healthy group (D, *n* = 10) (FDR *p* < 0.05, Table S5). Venn analysis indicated that over 80% of the changed miRNAs were unique to one of the comparisons, and none were common to all comparisons (Figure 3A, Table S5).

**Figure 4:**
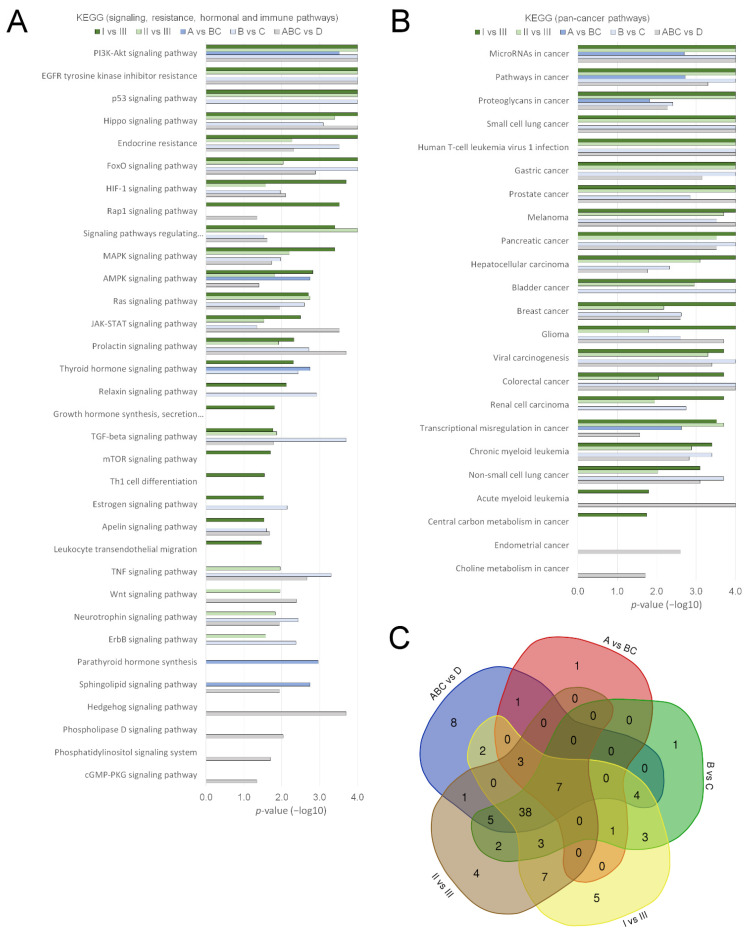
KEGG pathway analysis for differentially expressed miRNAs. The figure shows significant KEGG pathways for mRNAs targeted by the differential miRNAs from miRNAseq. (**A**) KEGG signalling, resistance, hormonal and immune pathways and (**B**) Pan-cancer associated pathways for comparison of prostate cancer (PCa) status and progression groups (A–C, I–III, *n* = 30 patients) and the healthy group (D, *n* = 10). (**C**) Venn diagram of all significant pathways for the comparisons. Differential miRNAs with FDR *p* or *p* < 0.05 were included in the analysis (Tables S5 and S10). Pathway *p*-values are shown in −log10 scale.

According to gene set enrichment analysis for KEGG pathways, the DE miRNAs from these status group comparisons (A vs. BC, B vs. C) targeted mRNAs from a comprehensive set of cancer and progression-linked signalling, resistance, hormonal and pan-cancer pathways (Figure 4A,B). A similar result was obtained from the ABC vs. D comparison. According to the numbers, we found 13 pathways between Gleason score ≥ 8 vs. 7 groups (A vs. BC), whereas the Gleason score of 7 status groups (B vs. C) differed in 64 pathways and PCa patients and the healthy group (ABC vs. D) by 69 pathways (*p* < 0.05, Table S7). However, overall >70% of the pathways from these comparisons overlapped with each other (Figure 4C). They included the EGFR tyrosine kinase inhibitor and the endocrine resistance pathways and the PI3K-Akt, p53, FoxO, HIF-1, JAK-STAT, TNF, TGF-beta, Wnt, MAPK, AMPK, Ras and Rap1 pathways, as well as the prolactin, neurotrophin, apelin and estrogen pathways (FDR *p* or *p* < 0.05, Figure 4A, Table S7). As expected, “miRNAs in cancer” and “prostate cancer” were among the top significant pathways (Figure 4B). In addition, the targeted pathways included apoptosis, cellular senescence and choline metabolism as well as various viral pathways (Table S7).

We next performed qPCR to confirm the expression of selected DE miRNAs (*n* = 32 totally for all comparisons between groups A–C or A–C vs. D) and reference candidate miRNAs (*n* = 10, see methods) using all the samples. Here, in addition to miRNAs from the main comparisons (Table S5), we included some DE miRNAs from additional comparisons (Table S6), such as members of the miRNA 888-cluster—miR-891b, -888-5p, -892a, -892b, -891a-5p—differing particularly in comparisons that included group B (*p* < 0.05). In the PCA of the qPCR, despite some tendency of separation between PCa and healthy, a clear clustering of the status groups was not found (Figure S3C).

**Figure 5:**
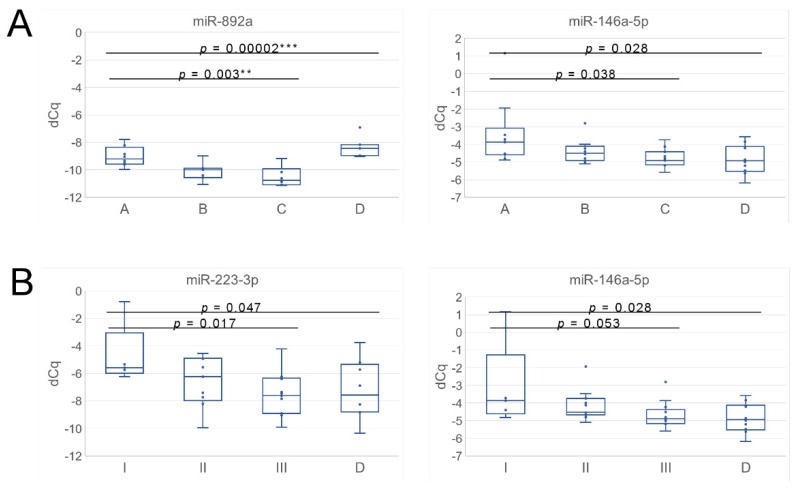
Confirmation of differentially expressed miRNAs by qPCR. Figure depicts expression of the miRNAs with the most significant changes between (**A**) PCa status groups (A–C) or (**B**) PCa progression groups (I–III). For clarity, only ANOVA *p*-values are shown with and without the healthy (D) group (for individual comparisons, see Table 2). Box-and-whiskers plots present the first quartile, median and third quartile, minimum and maximum values and outliers that are marked outside the whiskers range. Stars after *p*-values indicate the false discovery rate (FDR *p*-value ** < 0.01, *** < 0.001). Delta quantification cycle (dCq).

**Figure 6:**
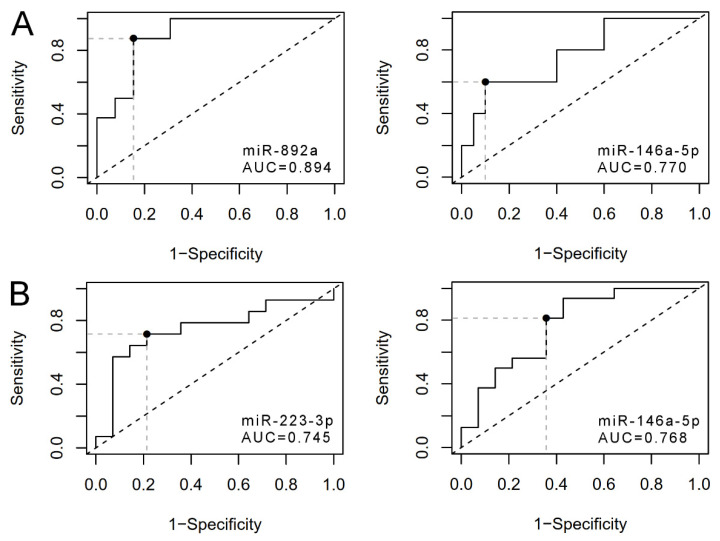
ROC curves. Receiver operator characteristic (ROC) curve analysis using qPCR data of the key differentially expressed miRNAs: (**A**) miR-892a and miR-146a-5p for separating Gleason score ≥ 8 (Group A) from the Gleason score 7 (B and C combined), as well as (**B**) miR-223-3p and miR-146a-5p for separating progressors (Groups I and II combined) from non-progressors (Group III). Area under the curve (AUC).

For the comparison of status groups A, B and C, combined or individually, two miRNAs, miR-146a-5p and -892a, were significantly changed similarly to miRNAseq (FDR or *p* < 0.05 for pair-wise comparisons and ANOVA, Figure 5A, Table 2). The qPCR analysis additionally suggested dysregulation (1.3–4.2 fold) of miR-34a-5p, -424-5p, -892b, -1299 and -3065-5p between some of the groups, A–C (*p* < 0.05, Table S8), and 15 miRNAs between ABC vs. D, with the same changes as in the miRNAseq (FDR or *p* < 0.05, Tables S8 and S9). For nine of the 15 miRNAs, qPCR suggested changes also between some PCa groups, including miR-892a. We then conducted a ROC curve analysis using the qPCR data of the two most DE miRNA. Hsa-miR-892a, as a standalone marker for differentiating A from BC, produced an area under the curve of 0.894 (95% confidence interval 0.758–1.031, *p* = 1.46 × 10^−8^, Figure 6A), as well as 0.846 (0.546–0.981) specificity and 0.875 (0.473–0.997) sensitivity for the optimal cut-point. Similarly, hsa-miR-146a-5p produced an area under the curve of 0.770 (95% confidence interval, 0.587–0.953, *p* = 0.004, Figure 6A), as well as 0.900 (0.683–0.988) specificity and 0.600 (0.262–0.878) sensitivity for the optimal cut-point.

### 3.4. Analysis of Prostate Cancer Progression Groups Uncovered Unique miRNA Signatures and Overlapping Cancer Progression-Linked Pathways

To understand which of the miRNAs in the uEV samples were linked to disease progression, we reanalysed the miRNAseq data using disease progression groupings formed based on clinical information 4–7 years post-RP (Table 1). Five patients had the most progressive disease during the follow-up time (PCa I group). Most of them (4/5) came from status group A (Table 1), including P30. In miRNAseq PCA, the P30 sample was among the non-clustering samples (Figure S3D), whereas in PCA of the qPCR data, it clustered together with other samples (Figure S3E). PCa group II included 3–4 patients from all status groups A–C (Table 1). Interestingly, the non-progressing group III also included patients from all the status groups, although eight out of 14 came from Group C (Table 1). Thus, the prior PCa status did not directly dictate the PCa progression.

In total, 151 unique miRNAs were DE between the PCa progression groups in the miRNAseq data (FDR *p* or *p* < 0.05, Table S10). Out of these, 59 (39%) and 66 (44%) miRNAs were DE between the most (I and II) compared to the least (III) progressed groups, respectively. Groups I and II differed in the expression of a lower number of miRNAs—23 (15%). Again, the proportion of miRNAs uniquely DE in different comparisons between progression groups was overall high—74% of the total (Figure 3B). Interestingly, the miRNAs were also mostly different from those found in the status group analysis (Figure 3C).

Despite the unique miRNAs, analysis for KEGG pathways again revealed most of the same cancer and progression-linked pathways found from previous comparisons between the PCa status groups or the PCa vs. healthy group (FDR *p* or *p* < 0.05, Table S11, Figure 4). Particularly, both progressed groups I and II differed from Group III of the non-progressors by 14 commonly known or potential cancer associated signalling, resistance and hormonal pathways (Figure 4A), and altogether they shared 58 differential pathways (Figure 4C). The pathways that differed uniquely only between Groups I and III, and not between other progression groups, included some hormonally regulated pathways such as those of relaxin, growth hormone, apellin and estrogen, as well as Rap1, mTOR and leukocyte pathways (Figure 4A). Notably, out of all the comparisons, we found growth hormone, mTOR and leukocyte pathways here only. Group II differed uniquely from group III by Wnt, TNF, ErbB and neurotrophin terms. Analysis of KEGG pathways from other comparisons gave no pathways (I vs. II or I + II vs. III) or only one (I vs. II + III) significant cancer associated pathway (Table S11).

To further confirm that the DE uEV miRNAs between progression groups were cancer-pathway regulating miRNA, we carried out overrepresentation analysis with the TAM 2.0 tool [33,34]. It confirmed that 32–61% of the DE miRNAs from all comparisons were identified as PCa related and overrepresented by 2.2–3.8-fold (Bonferroni adj *p* or FDR *p* < 0.01, Table 3). The top significant functions of the miRNAs differentiating groups I and II from III were linked to, e.g., epithelial-to-mesenchymal transition (EMT), inflammation, hematopoiesis and apoptosis (Bonferroni adj *p* or FDR *p* < 0.01). In addition, I differed from III (FDR *p* < 0.01) in the angiogenesis pathway. However, again no highly significant functions were found for the comparisons of I vs. II, or I and II combined vs. III.

Two of the 18 selected DE miRNAs from miRNAseq were confirmed by qPCR: miR-223-3p and -146a-5p (*p* < 0.05 for pairwise comparisons and/or ANOVA, Figure 5B). Both showed upregulation in the most progressed groups I and II relative to Group III (Table 2). In addition, qPCR suggested changes in 12 more miRNAs between some of the progression groups, including seven (miR-15a-5p, -16-5p, -31-3p, -31-5p, 92a-3p, -210-3p, 3065-5p) that were also DE between PCa status groups and/or the healthy group (Tables S8 and S9). ROC curve analysis using the qPCR data for differentiating progressors I and II from non-progressors III showed AUC 0.745 for miR-223-3p (*p* = 0.016) with a sensitivity of 0.714 (95% ci, 0.571–0.857) and a specificity of 0.786 (0.643–0.929) at the optimal cut-point (Figure 6B). The AUC for miR-146a-5p was 0.768 (*p* = 0.003) with a sensitivity of 0.812 (0.544–0.960) and a specificity of 0.643 (0.351–0.872) in the optimal cut-point (Figure 6B). Together, the findings suggested that uEV from different PCa status and progression groups display miRNAs regulating comprehensive and largely overlapping sets of cancer pathways. Despite the overlap, the individual miRNA signatures differentiating each of the PCa status or progression groups were unique.

### 3.5. Correlation Study of miR-146a-5p, -892a and -223-3p Targets in Patient EV Reveals mRNAs of Interest for Detecting Prostate Cancer Progression

As the DE miRNAs showed rather unique signatures in different PCa groups, we performed a small, personalized correlation study to decipher the expression of the key miRNAs and their potential mRNA targets in matched uEV and plasma EV (pEV) samples from three patients with advanced status (group B, P33) or metastatic disease (group E, P34 and 35, Table 4). The EV quality was monitored with Western blotting, EM, total RNA profiles in Bioanalyzer Pico assays and sequencing using either the correlation study samples or healthy control samples collected and processed the same way (Figure S4). UEV quality appeared similar to before [4,7,30] and to that of pEV in previous publications using ExoEasy [45,46].

We started the study by sequencing miRNAs in the samples from P33 with GS 7 disease, from whom we had collected urine and plasma pre- and post-RP. A healthy male (HC11, group D) and female donor (HC13) were included as technical or biological controls. In addition to uEV and/or pEV, we included plain plasma in this analysis. All samples passed the basic miRNAseq quality evaluation (Phread scores > 30) and resulted in an average of 0.9 M reads (range, 0.7–1.2 M, Table S12) and detection of 501, 407 and 633 miRNAs from uEV, pEV and plasma, respectively. MiRNA-146a-5p showed consistently higher expression in all of the pre-RP samples compared to post-RP samples or HC samples, with the exception of pEV of HC11 (Figure S4). MiR-223-3p gave similar results from pEV and plasma, but it was not detected in the uEV samples. MiR-892a could not be detected in any sample using this platform.

We next carried out mRNAseq to explore which mRNA targets of miR-146a-5p, -892a and -223-3p were particularly expressed in the uEV or pEV of the three patients (Table 4). All uEV samples, including controls, i.e., the post-RP sample of P33, HC11 (3 technical replicates) and HC13 used in quality control (Figure S4), produced an average of 23.5 M reads mapping to transcriptome (range, 15.8–31.1 M) and 19,185 expressed mRNAs (>0 CPM, range, 18,128–20,620, Table S13). The corresponding values from pEV were 11.2M transcriptome reads (range, 6.0–17.9 M) and 18,866 mRNAs (>0 CPM, range, 11,132–28,210, Table S13). For uEV, maximally only 3% of reads came from rRNA and mitochondrial RNA combined, while for pEV their proportion was much higher, ranging between 13% and 43% (Table S13).

The mRNAs were then compared with mRNA target lists for miR-146a-5p (*n* = 434), -892a (*n* = 427) and -223-3p (*n* = 370), obtained from miRWalk (Table S14). As 80%, on average, of the targets were expressed in any EV sample (Table S13), we restricted our analysis to mRNAs expressed in at least a 5-fold higher level in the primary patient EV samples than in the post-RP sample of P33, thereby attempting to focus on detectable transcripts from prostate, PCa or metastasis. As positive controls for this strategy, we checked the expression of SPDEF, a known uEV biomarker with roles in PCa initiation and progression [47] and TGM4, detected in uEV with enriched expression in prostate and PCa tissues and correlated with unfavourable prognosis [19,48]. The expression of SPDEF and TGM4 mRNA were >5-fold higher in all three primary PCa patient uEV samples, relative to the post-RP uEV sample (Table S4). Conducting this analysis for the three miRNA-targeted mRNAs, we found a total of 217 mRNAs in uEV and 269 mRNAs in pEV (Table S15). The expressed targets included, e.g., >10 solute carriers and protocadherins, few families with sequence similarity members (FAM9C, -13C, -98B, -160B1), cytokine signalling related mRNAs (IL6ST, IL1RL2), neuro-oncological ventral antigens 1 and 2 (NOVA 1 and 2) and many other genes of interest in PCa pathology, such as CDON, DPY19L2, FNLA, L1CAM, MMP16, NLRP3, NRP2, PAX5, STARD4, STXBP5L, SULT1B1, VCAN and SYNPO2. For uEV and pEV, respectively, we found 40 and 45 genes common to all three patients, 13 and 59 for P33 only (Group B) and 145 and 102 for P34 and P35 (group E), together or alone. There were 70 common targets between the lists of uEV and pEV (in Table S15), out of which 46 appeared systematically in both uEV and pEV from the same patient (Table 5).

As the miR-892a in particular had showed some differences in expression levels between status groups ABC and group D in the main study (Table S9), we additionally compared the expression of mRNA targets in the patient EV relative to HC11 EV (Table S16). We divided the mRNAs according to them showing a binary expression pattern (expressed in primary PCa samples, but not in post-RP samples of P33 or in HC11 samples), or mRNAs upregulated ≥5-fold relative to post-RP and ≥5-fold up- or downregulation relative to HC11. However, miR-892a targets in uEV were found in this analysis as often as the targets of miR-146a-5p and -223-3p (~11% of the listed mRNA targets for all three miRNAs). Thus, the correlation study suggested that uEV and pEV offer both unique and overlapping contents for studying both miRNA and their target mRNA in PCa pathogenesis.

## 4. Discussion

The quest for detecting or predicting aggressive prostate cancer and its progression has been ongoing for decades. The most sought-after goal involves non-invasive biomarkers, in which regard urine and, lately, uEV in particular have been investigated [8,11,49]. However, despite the interest, a complete picture is missing regarding cancer pathways that could be detected via uEV. Our analysis of uEV miRNA in prostate cancer status and progression groups, as well as target pathways and mRNAs, adds new insight regarding EV as a liquid biopsy.

Our results from uEV pointed to changes in unique sets of miRNAs in different PCa status and progression groups. These included several miRNAs that have been previously reported to regulate processes contributing to PCa development and metastasis, including both tumour suppressive and promoting roles in primary tumour—cancer associated fibroblasts, extracellular matrix, angiogenesis, EMT—and in migration, extravasation and colonization of metastatic sites [50]. Our pathway analysis consistently indicated that the DE miRNAs regulated the majority of the most important cancer progression promoting pathways and processes (Figure 4, Table 3). This was evident when comparing both PCa status and progression groups and also between PCa and healthy groups, which supports the idea that uEV offer a good sample for studying miRNAs from these pathways. The high number of shared pathways was still a surprising finding given the unique miRNA signatures (Figure 3 and Figure 4). The findings therefore suggests that specific miRNA signatures regulating the common pathways may uniquely associate with specific PCa disease and progression stage.

Pathways showing changes in all or most comparisons, and are therefore robustly associated with a higher Gleason score, advanced status or progression, included receptor tyrosine kinase –linked pathways (EGFR, PI3K-Akt, Ras, MAPK, JAK-STAT), p53, and the TGF-beta, AMPK and HIF-1 pathways. All these pathways have been linked to PCa progression and bone metastasis [51]. More unique changes were found in the mTOR and immune/leukocyte pathways (in I or II vs. III), several hormonal pathways (commonly changed in I vs. III) as well as the Wnt, TNF, ErbB and neurotrophin pathways (II vs. III or B vs. C, Figure 4, Table 3). Here, the association between the leukocyte pathways and aggressive progression is of interest, because infiltration of some T-cell subtypes has been shown to associate with a higher risk of PCa-induced death [52]. Out of the hormonal pathways, relaxin and apelin upregulation have been linked to metastasis or androgen independence [53,54]. For instance, Thompson et al. found that relaxin expression increased in RP specimens obtained after 6 months of androgen ablation, in androgen independent tumours and in bone metastases [53]. Local estrogen signalling may connect to PCa progression and development of hormone refractory disease [55]. Finally, previous work suggests that neurotrophins are particularly expressed in metastatic PCa, and—with EGF signalling—can mediate autocrine signalling, which is important for the progression of PCa [56]. In our results, brain-derived neurotrophic factor (BDNF) appeared as the target neurotrophic factor of miR-204-5p, downregulated upon progression (Tables S10 and S11). BDNF was also higher expressed in the pEV sample of P35 compared to the controls (Table S4). In agreement, loss of miR-204 results in BDNF/TRKB overexpression and activation of the Akt/mTOR/Rac1 signalling pathway, cancer cell migration and invasion in many cancers [57]. In PCa, overexpressed BDNF promotes progression via induction of EMT and anoikis resistance [58]. Early data suggests that neurotrophins induce neuregulin 1 (NRG1) release [59]. Interestingly, the neuregulin 1 secreted from cancer associated fibroblasts was recently shown to promote antiandrogen resistance [60]. This links our neurotrophin pathway finding to a possible PCa resistance mechanism. As neuregulin activates the HER3 and then the PI3K/Akt pathway [60,61], some additional pathways found in our study (e.g., PI3K/Akt signalling) could be associated to this chain of events. Thus, the EV results warrant further studies in men with advanced PCa treated with novel antiandrogens.

On the level of single miRNAs, the best candidates—miRNA-892a, miRNA-223-3p and miRNA-146a-5p—were all upregulated in patients with GS ≥ 8 or in patients progressing via the most aggressive disease course post-RP (Figure 5 and Figure 6). In agreement with this, upregulation of the miRNA-888 cluster was previously shown in expressed prostatic secretion (EPS) urine exosomes and PC3 cell lines from high-grade prostate cancer and downregulation in lower grade cancer [15]. Upregulation of miRNA-223-3p was demonstrated in prostate cancer tissues and cell lines and was found to target SEPTIN-6 [62]. However, SEPTIN-6 mRNA expression appeared steady in all patients and EV types in our correlation study (Table S4). Both miR-223-3p and miR-888 clusters were dysregulated in semen EV from PCa patients relative to healthy controls [63]. Even if also upregulated in benign prostate hyperplasia, a combination of miR-223-3p with two other miRNAs and PSA was able to discriminate PCa patients from hyperplasia controls [63,64].

Interestingly, miR-888 cluster and miRNA-223-3p reside within X-chromosome, where particularly Xq27–28 within the HPCX1 locus has been associated with hereditary PCa and susceptibility to PCa, especially in Finland [15,65,66]. The study by Mattila et al. [66] screened Finnish families with a strong linkage to hereditary PCa and to this locus: miR-223 and also miR-146a (chromosome 5) were dysregulated in the lymphoblastoid cells from patients with hereditary PCa compared to healthy controls. Hence, we may have identified these particular miRNAs as we studied a cohort from a Finnish biobank.

Even if dysregulation of miR-146a has been widely linked to cancer, its upregulation in uEV may appear contradictory to its tumour suppressive functions and downregulation in PCa tissues [50,67,68,69,70]. For example, miR-146a-5p downregulation was observed in castration resistant PCa tissues compared to androgen dependent PCa tissues, and its upregulation in PCa cells inhibited anchorage-independent growth, migration, invasion and angiogenesis via targeting the EGFR pathway or Rac1 [68,70]. The results from uEV and tissues/cells may differ, because uEV could serve as a disposal route for some tissue components, thus decreasing their quantity in tissues. Disposing of tumour suppressors could bring a growth advantage to PCa cells [71]. In support of this, Zhang et al. observed this kind of a difference in the tumour suppressor miR-15a-5p levels of hepatocellular carcinoma EV and cells [72]. Additionally, in the case of mRNAs, the transcripts in parental cells and their EV may differ [73]. However, prior data from urine has shown downregulation of miR-146a-5p [13]. The contradictory results could be explained by the different sample type (urine vs. uEV) and compared groups: we compared PCa status and progression groups, while the other study compared PCa to benign prostate hyperplasia patients [13]. The results could equally well differ due to choices concerning any (pre)analytical steps—standardization of methods is missing, particularly for uEV [5,7,20,24,64].

Our study targeted miRNAs and mRNAs that were detected in small uEV-enriched samples isolated by ultracentrifugation, or pEV samples isolated with ExoEasy without further steps of purification or enzymatic treatments (RNAse/proteinase). These steps attempt to remove miRNA in other carriers than EV or miRNA associated with the molecular corona on the EV surface [23,27,74,75]. Our detected miRNAs can therefore be located in or on EV or in other small-sized miRNA carriers remaining in the samples, including recently identified supermeres [27,76]. Other limitations of the study include relatively small sample numbers in the individual groups, which affects statistical power, and modest fold changes for some miRNAs. We had limited success in confirmation of the DE miRNAs by qPCR in the main study or by using another miRNAseq platform in the correlation study. While the minute quantities of RNA in EV pose a challenge for miRNAseq, the qPCR validation problem may be due to lack of standard reference miRNAs for uEV and PCa studies. Thus, even if we strived to identify stable miRNAs in our and previous datasets, currently accumulating knowledge of uEV miRNAs hopefully leads to identification of better, widely accepted reference miRNAs in the future [20,21].

The complexity of EV miRNA research for the notoriously heterogeneous PCa could be helped by conducting larger or personalized studies targeting not only miRNA but also the mRNAs within EV. In this regard, our simple correlation study of three patients uncovered a notable number of mRNA targets for miR-146a-5p, -223-3p and -892a in uEV and pEV. This approach appears promising based on the high number of genes with previous implications in PCa pathogenesis. For example, PCa patient EV expressed genes dysregulated or altered in PCa and/or metastasis (e.g., FNLA, MMP16, CDON, NRP2, PAX5, SULT1B1, L1CAM, SCHBP1) [77,78,79,80,81,82,83,84], in castration resistant prostate cancer (DPY19L2, NOVA1, NOVA2) [85,86] and in enzalutamide resistant (SLC6A15) [87] or androgen independent PCa cells (CALN1) [88]. They also expressed tumour suppressors (e.g., SULF1, EBF3) [89,90], many protocadherin family members, which have both suppressor and oncogenic roles in PCa [91], and solute carrier family members (e.g., SLC35F1, SLC26A2) implicated in drug uptake and efficacy modulation [92]. It is clear that the significance of their EV expression in PCa remains to be elucidated—some of the targets were expressed at low levels. However, as we discovered interesting, but only partly overlapping, targets from the matched uEV and pEV (Table 5 and Table S15), our study may help to focus future efforts with multiple patient and control types to the best EV source for selected miRNAs, mRNAs or their integrative analysis.

## 5. Conclusions

In conclusion, this study revealed that uEV samples contain miRNAs regulating well-known and emerging cancer pathways across the axis from cancer development to metastasis and to therapy resistance. We further identified cancer status and progression-associated miRNAs that were located in the X-chromosome and/or had been previously linked to hereditary prostate cancer, especially among Finns. As patient EV expressed a notable number of PCa-associated mRNA targets for these key miRNAs, EV appear to provide candidate prognostic biomarkers to be further explored in large, personalized medicine trials.

## Figures and Tables

**Table 1:** Clinical characteristics of groups in the main study. Data of prostate cancer patients in the status Groups A–C or healthy technical controls, D, or the same patients classified into prostate cancer progression Groups I–III after 4–7 years follow-up. Numbers in parenthesis denote the subject number for whom the information was available—in the absence of parenthesis, the information was available for all patients. After prostatectomy (post-RP), prostate-specific antigen (PSA).

Status Groups	A	B	C	D	ANOVA *p*-Value (A–C)	Progression Groups	I	II	III	ANOVA *p*-Value (I–III)
Number of subjects	10	9	11	10		Number of subjects	5	11	14	
						Previous classification in status group (number of subjects)				
						A	4	4	2	
						B	1	4	4	
						C	0	3	8	
Age (years)					0.209	Age (years)				0.777
Mean	69	64	63	<45		Mean	67	65	64	
Range	54–74	51–75	51–73			Range	62–74	51–75	54–73	
Gleason score (number of subjects)					2.1 × 10^16^	Gleason score (number of subjects)				0.121
7	0	9	11			7	1	7	12	
3 + 4	0	3	4			3 + 4	0	5	2	
4 + 3	0	6	7			4 + 3	1	2	10	
8	2	0	0			8	2	0	0	
4 + 4	2	0	0			4 + 4	2	0	0	
9	8	0	0			9	2	4	2	
4 + 5	8	0	0			4 + 5	2	4	2	
10	0	0	0			10	0	0	0	
Stage (number of subjects)					0.005	Stage (number of subjects)				0.026
T2	3	3	10			T2	1	4	11	
T3	7	6	1			T3	4	7	3	
Pathological features-prostatectomy tissues (number of subjects)						Pathological features-prostatectomy tissues (number of subjects)				
Positive surgical margin	3	7	0			Positive surgical margin	2	5	3	
Growth through capsule	6 (9)	6	1			Growth through capsule	3 (4)	7	3	
Invasion to seminal vesicles	3 (9)	1 (8)	0			Invasion to seminal vesicles	2 (3)	1	1	
Lymph node positivity	1 (8)	2 (7)	0 (4)			Lymph node positivity	2 (4)	0 (6)	1 (9)	
PSA (post-RP) (number of subjects or concentration)					0.260	PSA (post-RP) (number of subjects or concentration)				0.017
<0.05 (ng/mL)	5	5	8			<0.05 (ng/mL)	0	5	13	
≥0.05, <0.2, (ng/mL)	2	2	3			≥0.05, <0.2, (ng/mL)	1	5	1	
≥0.2, (ng/mL)	3	2	0			≥0.2, (ng/mL)	4	1	0	
Range for ≥0.05 (ng/mL)	0.12–8.42	0.05–0.53	0.06–0.08			Range for ≥0.05 (ng/mL)	0.14–8.42	0.05–0.53	0.08	
						Events and treatments during follow-up (number of subjects)				
						Death due to Pca	2	0	0	
						Metastasis (M1)	3	0	0	
						Hormonal treatment	5	0	0	
						Secondary treatment	5	10	0 (10)	
						Biochemical recurrence	5	6	0	

**Table 2:** Fold changes of differentially expressed miRNAs between prostate cancer groups in qPCR. Table includes only the most significant miRNAs with statistically significant changes in both miRNAseq analysis and in qPCR validation. Results marked in bold had a higher statistical significance (FDR *p* < 0.05 to <0.0001) than the rest (*p* < 0.05 to <0.0001). Fold change (FC), quantitative polymerase chain reaction (qPCR).

	MiRNA Name			
Comparison	miR-146a-5p	miR-892a	miR-223-3p	FC or *p*-Value
A vs. BC	2.3	2.4		FC
A vs. B		2.1		FC
A vs. C	2.6	2.8		FC
B vs. C				FC
AB vs. C	1.9	2.0		FC
ANOVA A–C	0.038	**0.003**		*p*-value
ANOVA A–D	0.028	**0.00002**		*p*-value
I vs. II				FC
I vs. III	3.0		6.9	FC
II vs. III	1.5, *p* = 0.051			FC
I vs. II + III			5.4	FC
I + II vs. III	1.8		3.0	FC
ANOVA I–III	0.053		0.017	*p*-value
ANOVA I–III and D	0.028	0.007	0.047	*p*-value

**Table 3:** Overrepresentation analysis using miRNAs differing between PCa progression groups. Table shows overrepresentation (fold) and miRNAs associating with the disease term “prostate cancer” and the most significant function term(s) (FDR *p* < 0.05, or *p* < 0.001, if no terms reached the FDR limit). Bonferroni adjusted *p*-value is also shown. Differentially expressed (DE).

Comparison	Category	Term	Count	Fold	*p*-Value	Bonferroni	FDR	DE miRNAs
I vs. II	Disease	Carcinoma, Prostate	6	2.2	NaN	0.0 × 10⁰	0.0 × 10⁰	382, 323a, 139, 22, 187, 485
	Function	Angiogenesis	5	7.2	4.4 × 10^−^^4^	1.4 × 10^−^^1^	8.7 × 10^−^^2^	891a, 382, 22, 184, 363
I vs. III	Disease	Carcinoma, Prostate	33	3.6	NaN	0.0 × 10⁰	0.0 × 10⁰	30d, 194-2, 1307, 135a-2, 138-1, 375, 497, 30c-1, 30a, 222, 378a, 196a-2, 1297, 195, 27b, 221, 4516, 503, 194-1, 135a-1, 138-2, 187, 1299, let-7c, 148a, 149, let-7e, 34c, 30c-2, 21, 141, 223, 29a
	Function	Epithelial-to-Mesenchymal Transition	19	6.5	7.9 × 10^−^¹²	8.4 × 10^−^⁹	9.4 × 10^−^¹⁰	486-2, 221, let-7c, 1246, 30d, 194-2, let-7e, 486-1, 194-1, 30c-1, 30a, 30c-2, 542, 21, 141, 223, 29a, 375, 192
	Function	Hematopoiesis	15	7.4	2.2 × 10^−^¹⁰	2.4 × 10^−^⁷	2.0 × 10^−^⁸	486-2, 221, let-7c, let-7e, 486-1, 30c-1, 30c-2, 222, 378a, 196a-2, 363, 223, 196a-1, 29a, 142
	Function	Angiogenesis	15	6.5	1.7 × 10^−^⁹	1.8 × 10^−^⁶	9.8 × 10^−^⁸	486-2, 891a, 221, 1246, 149, 486-1, 30a, 1275, 222, 378a, 10b, 363, 21, 27b, 497
	Function	Aging	14	6.3	1.1 × 10^−^⁸	1.2 × 10^−^⁵	5.5 × 10^−^⁷	221, let-7c, 148a, 30d, 194-2, let-7e, 194-1, 30a, 222, 10a, 21, 141, 223, 195
	Function	Inflammation	18	4.5	1.6 × 10^−^⁸	1.7 × 10^−^⁵	7.3 × 10^−^⁷	194-2, 99b, 135a-2, 138-1, 222, 27b, 192, 221, 194-1, 135a-1, 138-2, 148a, 34c, 21, 141, 223, 29a, 142
	Function	Osteogenesis	13	6.2	4.5 × 10^−^⁸	4.8 × 10^−^⁵	1.8 × 10^−^⁶	221, 106a, 194-2, 194-1, 34c, 30a, 222, 378a, 138-2, 21, 1297, 195, 138-1
	Function	Apoptosis	17	4.5	4.5 × 10^−^⁸	4.7 × 10^−^⁵	1.8 × 10^−^⁶	135a-2, 138-1, 497, 1246, 30a, 222, 10a, 195, 221, 4516, 135a-1, 138-2, let-7c, 148a, 34c, 21, 29a
	Function	Cell Cycle	14	4.8	4.6 × 10^−^⁷	4.9 × 10^−^⁴	1.3 × 10^−^⁵	221, 503, 34c, 222, 138-2, 141, 196a-2, 21, 223, 196a-1, 195, 138-1, 27b, 497
	Function	Cell Proliferation	13	4.6	2.0 × 10^−^⁶	2.1 × 10^−^³	4.3 × 10^−^⁵	221, let-7c, 503, let-7e, 509-1, 34c, 222, 378a, 21, 509-2, 509-3, 29a, 27b
	Function	Immune Response	13	4.0	1.0 × 10^−^⁵	1.1 × 10^−^²	1.7 × 10^−^⁴	486-1, 30a, 196a-2, 27b, 192, 532, 196a-1, 486-2, 148a, 34c, 21, 223, 29a
	Function	Brain Development	8	6.3	2.2 × 10^−^⁵	2.3 × 10^−^²	3.3 × 10^−^⁴	221, 106a, 135a-1, 222, 10a, 10b, 135a-2, 192
	Function	T-helper 17 Cell Differentiation	6	8.9	3.0 × 10^−^⁵	3.1 × 10^−^²	4.1 × 10^−^⁴	30c-2, 141, 106a, 27b, 21, 30c-1
	Function	Pancreas Development	3	28.2	4.3 × 10^−^⁵	4.5 × 10^−^²	5.6 × 10^−^⁴	let-7e, 375, 30d
	Function	Cell Death	11	4.0	5.6 × 10^−^⁵	5.9 × 10^−^²	6.9 × 10^−^⁴	221, let-7c, 30d, let-7e, 30c-1, 30c-2, 222, 10b, 21, 29a, 497
	Function	Regulation of Stem Cell	11	3.9	6.3 × 10^−^⁵	6.6 × 10^−^²	7.8 × 10^−^⁴	221, 106a, 148a, 222, 10a, 21, 141, 223, 195, 142, 192
	Function	Myogensis	4	14.1	9.0 × 10^−^⁵	9.6 × 10^−^²	1.1 × 10^−^³	135a-1, 222, 135a-2, 221
	Function	Lipid Metabolism	8	5.0	1.2 × 10^−^⁴	1.3 × 10^−^¹	1.4 × 10^−^³	378a, 10b, 196a-2, 196a-1, 29a, 375, 27b, 192
	Function	Cleavage Stage Development	3	21.2	1.7 × 10^−^⁴	1.8 × 10^−^¹	1.8 × 10^−^³	375, 21, 34c
	Function	Nephrotoxicity	5	8.3	2.1 × 10^−^⁴	2.3 × 10^−^¹	2.2 × 10^−^³	30a, 30d, 29a, 192, 21
	Function	Onco-MiRNAs	7	5.3	2.2 × 10^−^⁴	2.4 × 10^−^¹	2.3 × 10^−^³	221, 106a, 194-2, 194-1, 222, 196a-2, 196a-1
	Function	Oxidative Stress	4	11.3	2.6 × 10^−^⁴	2.7 × 10^−^¹	2.6 × 10^−^³	503, 222, 21, 141
	Function	Smooth Muscle Cell Proliferation	5	7.8	2.9 × 10^−^⁴	3.0 × 10^−^¹	2.8 × 10^−^³	222, 138-1, 10a, 138-2, 21
	Function	Tumour Suppressor MiRNAs	9	3.9	3.3 × 10^−^⁴	3.5 × 10^−^¹	3.1 × 10^−^³	let-7c, let-7e, 34c, 138-2, 141, 195, 29a, 138-1, 27b
	Function	Cell Migration	4	10.3	3.9 × 10^−^⁴	4.2 × 10^−^¹	3.7 × 10^−^³	142, 509-3, 509-1, 509-2
	Function	Adipocyte Differentiation	7	4.8	4.4 × 10^−^⁴	4.6 × 10^−^¹	3.8 × 10^−^³	221, let-7e, 222, 378a, 375, 27b, 192
	Function	Adipogenesis	5	7.1	4.9 × 10^−^⁴	5.2 × 10^−^¹	4.2 × 10^−^³	148a, 194-2, 29a, 194-1, 363
	Function	Innate Immunity	7	4.7	5.1 × 10^−^⁴	5.4 × 10^−^¹	4.3 × 10^−^³	let-7c, 149, let-7e, 30a, 21, 223, 142
	Function	Skeletal Muscle Cell Differentiation	5	6.4	7.9 × 10^−^⁴	8.4 × 10^−^¹	6.1 × 10^−^³	30d, 30a, 542, 138-2, 138-1
	Function	Cholesterol Efflux	4	7.5	1.5 × 10^−^³	1.0 × 10⁰	9.3 × 10^−^³	486-2, 486-1, 27b, 378a
	Function	Regulation of Akt Pathway	5	5.4	1.8 × 10^−^³	1.0 × 10⁰	1.1 × 10^−^²	221, 222, 196a-2, 141, 196a-1
	Function	T-Cell Differentiation	4	7.1	1.9 × 10^−^³	1.0 × 10⁰	1.1 × 10^−^²	let-7e, let-7c, 10a, 21
	Function	Cardiotoxicity	4	6.6	2.4 × 10^−^³	1.0 × 10⁰	1.4 × 10^−^²	34c, 486-2, 486-1, 187
	Function	Glucose Metabolism	5	5.0	2.5 × 10^−^³	1.0 × 10⁰	1.4 × 10^−^²	let-7c, let-7e, 223, 195, 375
	Function	Cell Differentiation	7	3.5	3.0 × 10^−^³	1.0 × 10⁰	1.7 × 10^−^²	let-7c, 194-2, 503, let-7e, 194-1, 34c, 222
	Function	Cholesterol Homeostasis	3	9.4	3.1 × 10^−^³	1.0 × 10⁰	1.7 × 10^−^²	223, 30c-2, 30c-1
	Function	Bone Regeneration	5	4.7	3.4 × 10^−^³	1.0 × 10⁰	1.9 × 10^−^²	221, 34c, 222, 196a-2, 196a-1
	Function	Tumour Cell Radiation Sensitivity	2	18.8	3.6 × 10^−^³	1.0 × 10⁰	1.9 × 10^−^²	223, 21
	Function	Hormone-mediated Signalling Pathway	7	3.4	3.6 × 10^−^³	1.0 × 10⁰	1.9 × 10^−^²	221, 30d, 363, 21, 223, 29a, 375
	Function	Circadian Rhythm	4	5.1	6.5 × 10^−^³	1.0 × 10⁰	3.1 × 10^−^²	194-2, 194-1, 29a, 192
	Function	Cardiomyocyte Proliferation	2	14.1	7.1 × 10^−^³	1.0 × 10⁰	3.3 × 10^−^²	222, 10a
	Function	Peritoneal Cavity Homeostasis	4	4.9	7.7 × 10^−^³	1.0 × 10⁰	3.4 × 10^−^²	148a, 30a, 497, 192
II vs. III	Disease	Carcinoma, Prostate	34	3.6	0.0 × 10⁰	0.0 × 10⁰	0.0 × 10⁰	96, 200c, 574, let-7d, 409, 449a, 135a-2, 375, 497, 155, 182, 195, 204, 424, 4516, 503, 218-2, 135a-1, 146a, 187, 381, 455, 483, let-7c, 148a, 149, 130b, 487b, 191, 21, 141, 218-1, 92b, 29a
	Function	Apoptosis	20	5.1	2.4 × 10^−^¹⁰	2.5 × 10^−^⁷	3.2 × 10^−^⁸	96, 449a, 135a-2, 497, 155, 10a, 182, 195, 204, 424, 4516, 218-2, 135a-1, 146a, let-7c, 148a, 216a, 21, 218-1, 29a
	Function	Inflammation	18	4.3	3.5 × 10^−^⁸	3.8 × 10^−^⁵	2.2 × 10^−^⁶	584, 20b, let-7d, 135a-2, 155, 182, 424, 218-2, 135a-1, 146a, 455, 148a, 130b, 21, 141, 218-1, 29a, 328
	Function	Epithelial-to-Mesenchymal Transition	14	4.5	8.5 × 10^−^⁷	9.1 × 10^−^⁴	3.4 × 10^−^⁵	let-7c, 200c, 450a-2, let-7d, 191, 211, 542, 450a-1, 21, 141, 424, 29a, 375, 155
	Function	Aging	12	5.1	1.5 × 10^−^⁶	1.6 × 10^−^³	5.6 × 10^−^⁵	96, let-7c, 200c, 148a, let-7d, 146a, 10a, 21, 141, 195, 204, 155
	Function	Cell Cycle	13	4.2	5.4 × 10^−^⁶	5.7 × 10^−^³	1.6 × 10^−^⁴	96, 200c, 503, 191, 182, 141, 21, 449a, 195, 424, 92b, 155, 497
	Function	Cell Differentiation	10	4.8	2.3 × 10^−^⁵	2.5 × 10^−^²	5.5 × 10^−^⁴	96, let-7c, 200c, 503, 218-2, let-7d, 182, 218-1, 424, 155
	Function	Brain Development	8	6.0	3.1 × 10^−^⁵	3.3 × 10^−^²	7.0 × 10^−^⁴	218-2, 191, 135a-1, 10a, 10b, 218-1, 135a-2, 155
	Function	Myofibroblast Differentiation	3	26.9	4.9 × 10^−^⁵	5.2 × 10^−^²	9.8 × 10^−^⁴	218-1, 218-2, 424
	Function	Hematopoiesis	9	4.3	1.7 × 10^−^⁴	1.8 × 10^−^¹	2.5 × 10^−^³	let-7c, 20b, 218-2, let-7d, 146a, 363, 218-1, 29a, 155
	Function	Cardiomyocyte Proliferation	3	20.2	1.9 × 10^−^⁴	2.0 × 10^−^¹	2.8 × 10^−^³	204, 424, 10a
	Function	T-Cell Differentiation	5	8.4	1.9 × 10^−^⁴	2.1 × 10^−^¹	2.7 × 10^−^³	let-7c, 10a, 155, let-7d, 21
	Function	Cardiotoxicity	5	7.9	2.7 × 10^−^⁴	2.8 × 10^−^¹	3.5 × 10^−^³	424, 146a, 1303, 182, 187
	Function	Nephrotoxicity	5	7.9	2.7 × 10^−^⁴	2.8 × 10^−^¹	3.5 × 10^−^³	let-7d, 29a, 130b, 200c, 21
	Function	O×idative Stress	4	10.8	3.1 × 10^−^⁴	3.3 × 10^−^¹	3.9 × 10^−^³	503, 146a, 21, 141
	Function	Cell Death	10	3.5	4.2 × 10^−^⁴	4.5 × 10^−^¹	5.0 × 10^−^³	let-7c, 130b, let-7d, 146a, 10b, 182, 21, 29a, 497, 155
	Function	Adipogenesis	5	6.7	6.1 × 10^−^⁴	6.5 × 10^−^¹	6.8 × 10^−^³	148a, 204, 455, 29a, 363
	Function	Toll-Like Receptor Signalling Pathway	3	13.5	9.0 × 10^−^⁴	9.7 × 10^−^¹	9.8 × 10^−^³	149, 146a, 381
	Function	Osteogenesis	8	3.7	1.2 × 10^−^³	1.0 × 10⁰	1.2 × 10^−^²	96, 200c, 218-2, 211, 21, 218-1, 195, 424
	Function	Neuron Differentiation	4	7.7	1.3 × 10^−^³	1.0 × 10⁰	1.3 × 10^−^²	218-1, 218-2, 96, 182
	Function	Regulation of Nf-Κb Pathway	3	11.5	1.5 × 10^−^³	1.0 × 10⁰	1.3 × 10^−^²	146a, 497, 21
	Function	Regulation of Stem Cell	9	3.1	2.0 × 10^−^³	1.0 × 10⁰	1.6 × 10^−^²	200c, 148a, 146a, 10a, 21, 182, 141, 195, 155
	Function	Cell Proliferation	9	3.0	2.2 × 10^−^³	1.0 × 10⁰	1.7 × 10^−^²	let-7c, 200c, 503, let-7d, 146a, 21, 449a, 29a, 92b
	Function	T-Cell Activation	3	10.1	2.4 × 10^−^³	1.0 × 10⁰	1.9 × 10^−^²	146a, 155, 21
	Function	Response to Estrogen	3	10.1	2.4 × 10^−^³	1.0 × 10⁰	1.9 × 10^−^²	146a, 21, 182
	Function	Embryonic Development	4	6.3	2.9 × 10^−^³	1.0 × 10⁰	2.2 × 10^−^²	20b, 130b, 10a, 21
	Function	Glucose Metabolism	5	4.8	3.1 × 10^−^³	1.0 × 10⁰	2.3 × 10^−^²	let-7c, let-7d, 625, 195, 375
	Function	Innate Immunity	6	3.8	3.8 × 10^−^³	1.0 × 10⁰	2.7 × 10^−^²	let-7c, 149, let-7d, 146a, 21, 155
	Function	Bone Regeneration	5	4.5	4.2 × 10^−^³	1.0 × 10⁰	2.7 × 10^−^²	20b, 130b, let-7d, 424, 155
	Function	T-helper 17 Cell Differentiation	4	5.7	4.4 × 10^−^³	1.0 × 10⁰	2.8 × 10^−^²	141, 20b, 155, 21
	Function	Granulopoiesis	3	8.1	4.9 × 10^−^³	1.0 × 10⁰	3.1 × 10^−^²	let-7d, 155, 21
	Function	Neurotoxicity	4	5.4	5.4 × 10^−^³	1.0 × 10⁰	3.3 × 10^−^²	92b, 96, 10a, 10b
	Function	Immune System(Xiao’s Cell 2010)	4	5.1	6.5 × 10^−^³	1.0 × 10⁰	3.9 × 10^−^²	20b, 146a, 363, 155
	Function	Cell Motility	4	5.1	6.5 × 10^−^³	1.0 × 10⁰	3.9 × 10^−^²	584, 130b, 10b, 21
	Function	Circadian Rhythm	4	4.9	7.7 × 10^−^³	1.0 × 10⁰	4.4 × 10^−^²	96, 191, 182, 29a
	Function	Cleavage Stage Development	2	13.5	7.8 × 10^−^³	1.0 × 10⁰	4.4 × 10^−^²	375, 21
	Function	Type II Pneumocyte Differentiation	2	13.5	7.8 × 10^−^³	1.0 × 10⁰	4.4 × 10^−^²	200c, 29a
	Function	Adiponectin Signalling	2	13.5	7.8 × 10^−^³	1.0 × 10⁰	4.4 × 10^−^²	218-1, 218-2
I vs. II + III	Disease	Carcinoma, Prostate	17	3.6	NaN	0.0 × 10⁰	0.0 × 10⁰	194-2, 500b, 376c, 132, 134, 378a, 139, 708, 29c, 503, 194-1, 187, 143, 1299, 483, 223, 29a
	Function	Inflammation	10	4.8	1.8 × 10^−^⁵	1.2 × 10^−^²	2.3 × 10^−^³	194-2, 144, 132, 134, 708, 194-1, 143, 140, 223, 29a
	Function	Adipogenesis	5	13.5	2.1 × 10^−^⁵	1.4 × 10^−^²	2.4 × 10^−^³	194-2, 140, 29a, 194-1, 29c
	Function	Circadian Rhythm	5	12.2	3.4 × 10^−^⁵	2.3 × 10^−^²	3.6 × 10^−^³	29c, 194-2, 194-1, 29a, 132
	Function	Epithelial-to-Mesenchymal Transition	8	5.2	8.6 × 10^−^⁵	5.9 × 10^−^²	5.3 × 10^−^³	29c, 194-2, 194-1, 542, 223, 29a, 144, 143
	Function	Hematopoiesis	6	5.7	4.7 × 10^−^⁴	3.2 × 10^−^¹	1.7 × 10^−^²	29c, 378a, 223, 29a, 144, 143
	Function	Cell Growth	2	35.9	9.9 × 10^−^⁴	6.7 × 10^−^¹	3.0 × 10^−^²	132, 143
	Function	Stress Response	2	26.9	2.0 × 10^−^³	1.0 × 10⁰	4.5 × 10^−^²	29c, 143
I + II vs. III	Disease	Carcinoma, Prostate	6	2.4	NaN	0.0 × 10⁰	0.0 × 10⁰	888, 323a, 134, 146a, 1299, 223
	Function	Regulation of Stem Cell	5	6.6	6.2 × 10^−^⁴	3.0 × 10^−^¹	1.1 × 10^−^¹	134, 146a, 223, 323a, 142

**Table 4:** Clinical characteristics of patients in the correlation study. Data of prostate cancer patients in the status groups B or E or a healthy technical control, D. Age is reported at the time of sample collection. Primary sample refers to pre-prostatectomy samples for P33 and samples obtained close to the time point of needle biopsy for P34 and P35. After prostatectomy (post-RP).

Individual	P33	P34	P35	HC11
Status group	B	E	E	D
Age (years)				
Primary sample	57	67	85	<45
Post-RP	58			
Stage				
	T3	T3-4	T3-4	
	N0M0	NXM1	NXM1	
Gleason score				
	7 (4+3)	7 (4+3)	8 (4+4)	
PSA (ng/mL)				
Primary sample	17	125	2.6	
Post-RP	0			

**Table 5:** List of mRNA targets expressed in matched uEV and pEV from the patients. Table includes 46 mRNA targets of miR-146a-5p, -223-3p or -892a that were higher expressed (≥5-fold) in the matched primary uEV and pEV samples from at least one PCa patient (P33–P35) than in the matched EV from a control (post-prostatectomy samples of P33). The higher expression per patient and EV type vs. respective control sample is marked with X.

mRNA Targets (miR-146a-5p, -223-3p or -892a)	uEV P33	uEV P34	uEV P35	pEV P33	pEV P34	pEV P35
MAP2	X	X	X	X	X	
SLC9A7	X	X	X	X		X
TLR2	X	X	X		X	X
LGSN	X	X	X		X	X
VWC2	X	X	X		X	X
STARD4	X	X		X	X	X
VCAN		X	X	X	X	X
FMNL3, FLNA	X	X	X	X		
ALG9, GDPD1	X	X	X		X	
CFTR, PKD2L2, POFUT2, ST8SIA1, SYNPO2, ZNF714	X	X	X			X
CADM2, MORC1, RGS5, SLCO3A1		X	X	X		X
HAL		X	X	X	X	
GABRB2		X	X	X		X
SULT1B1		X	X	X		X
INHBB	X			X	X	X
VNN1		X		X	X	X
ATG9A, RBL1, SPATA13, TSHZ3, XPR1			X	X	X	X
DSCC1	X	X		X		
NLRP3		X	X		X	
SLC35F1, VWA2		X	X			X
CTNNA2		X	X			X
SLC6A15		X	X		X	
STXBP5L	X			X		X
KCND3		X		X	X	
FZD1, TRDMT1, ZNF367			X	X		X
SHOX2		X			X	X
MDN1			X		X	X
IL1RL2, GJC1			X			X

## Data Availability

Data supporting the results are available as Supplementary Tables and Figures. The raw sequencing data analysed in this study is not publicly available due to local biorepository regulations. We provide raw miRNAseq and mRNAseq count data in the Tables S1, S3 and S4—the data from the healthy control group of the main study has been analysed in different context earlier [20].

## Supplementary Materials

The following are available online at https://www.mdpi.com/article/10.3390/cancers14030532/s1, Figure S1: Western blotting of uEV samples, Figure S2: Quality of miRNAseq, Figure S3: Principal component analysis of miRNAseq and qPCR data, Figure S4. Quality control and miRNA expression in the correlation study, Figure S5: Western blotting originals, Table S1: Raw count data from miRNAseq, Table S2: Quantitative PCR assay information for all assays and Normfinder stabilities in miRNAseq for the selected reference miRNAs, Table S3: Raw count data from miRNAseq in the correlation study, Table S4: Raw count data from mRNAseq in the correlation study, Table S5: Differentially expressed miRNAs between the main prostate cancer status groups and healthy controls from miRNAseq, Table S6: Additional comparisons of differentially expressed miRNAs between prostate cancer status groups and healthy controls from miRNAseq, Table S7: KEGG pathway analysis with differentially expressed miRNAs between prostate cancer status groups and healthy controls from miRNAseq, Table S8 Fold changes of differentially expressed miRNAs between prostate cancer groups in qPCR, Table S9: Fold changes of differentially expressed miRNAs between prostate cancer status groups and healthy controls in qPCR, Table S10: Differentially expressed miRNAs between prostate cancer progression groups from miRNAseq, Table S11: KEGG pathway analysis with differentially expressed miRNAs between prostate cancer progression groups from miRNAseq, Table S12: Read and miRNA counts in the miRNAseq of the correlation study, Table S13: Read, mRNA and miRNA targeted mRNA counts in the mRNAseq of the correlation study, Table S14: Target mRNAs of miR-146a-5p, -223-3p and -892a, Table S15: Expression of target mRNAs of miR-146a-5p, -223-3p and -892a in uEV and pEV from correlation study patients, Table S16: Binary, higher or lower expression of miR-146a-5p, -223-3p and -892a target mRNAs in uEV and pEV from correlation study patients compared to controls.
