# Supplementary material for: Exploration of Extracellular Vesicle miRNAs, Targeted mRNAs and Pathways in Prostate Cancer: Relation to Disease Status and Progression
Source: Cancers (Basel). 2022 Jan 21;14(3):532. doi: 10.3390/cancers14030532 (PMC8833493; doi:10.3390/cancers14030532)
Supplement: Supplementary file 1 [file cancers-14-00532-s001.zip › cancers-1463070-Supplementary Figures.pdf]

cancers

MDPI

Exploration of Extracellular Vesicle miRNAs, Targeted mRNAs and Pathways in Prostate Cancer: Relation to Disease Status and Progression

Maija Puhka, Lisse Thierens, Daniel Nicorici, Tarja Forsman, Tuomas Mirtti, Taija af Hällström, Elina Serkkola and Antti Rannikko

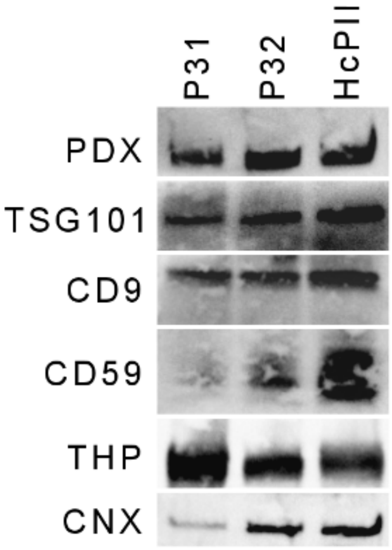

Figure S1. Western blotting of uEV samples. Western blotting of uEV enriched protein markers podocalyxin (PDX), TSG101, CD9 and CD59, as well as Tamm horsfall protein (THP) and calnexin (CNX) from urine samples collected and isolated similarly to the study samples. Healthy control pool II (HCpII), PCa patient samples (P31, P32), urinary extracellular vesicles (uEV).

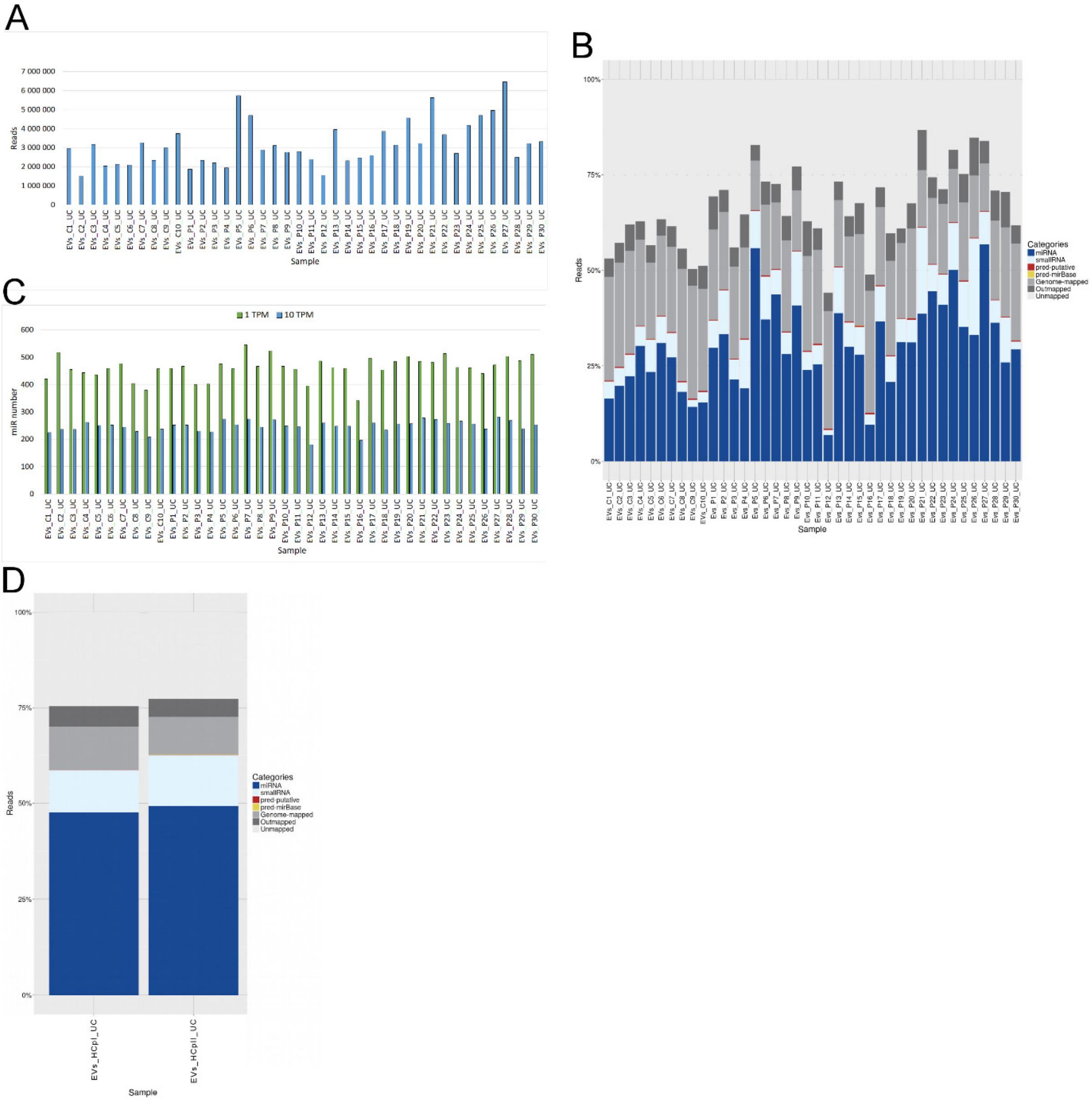

Figure S2. Quality of miRNAseq in uEV samples. Figure depicts (**A**) the number of unique molecular identifier corrected reads, (**B**) proportions of reads mapping to miRNA, other small RNA, predicted (pred) RNAs or elsewhere and, (**C**) the number of miRNAs detected in 1 or 10 transcripts per million (TPM) frequency for all samples included in the study. In (**D**), the reads mapping is shown for the two pools (HCpI and HCpII) used for uEV quality control in addition to the study samples (see Figure 2). Control (C), extracellular vesicles (EVs), patient (P), ultracentrifugation (UC).

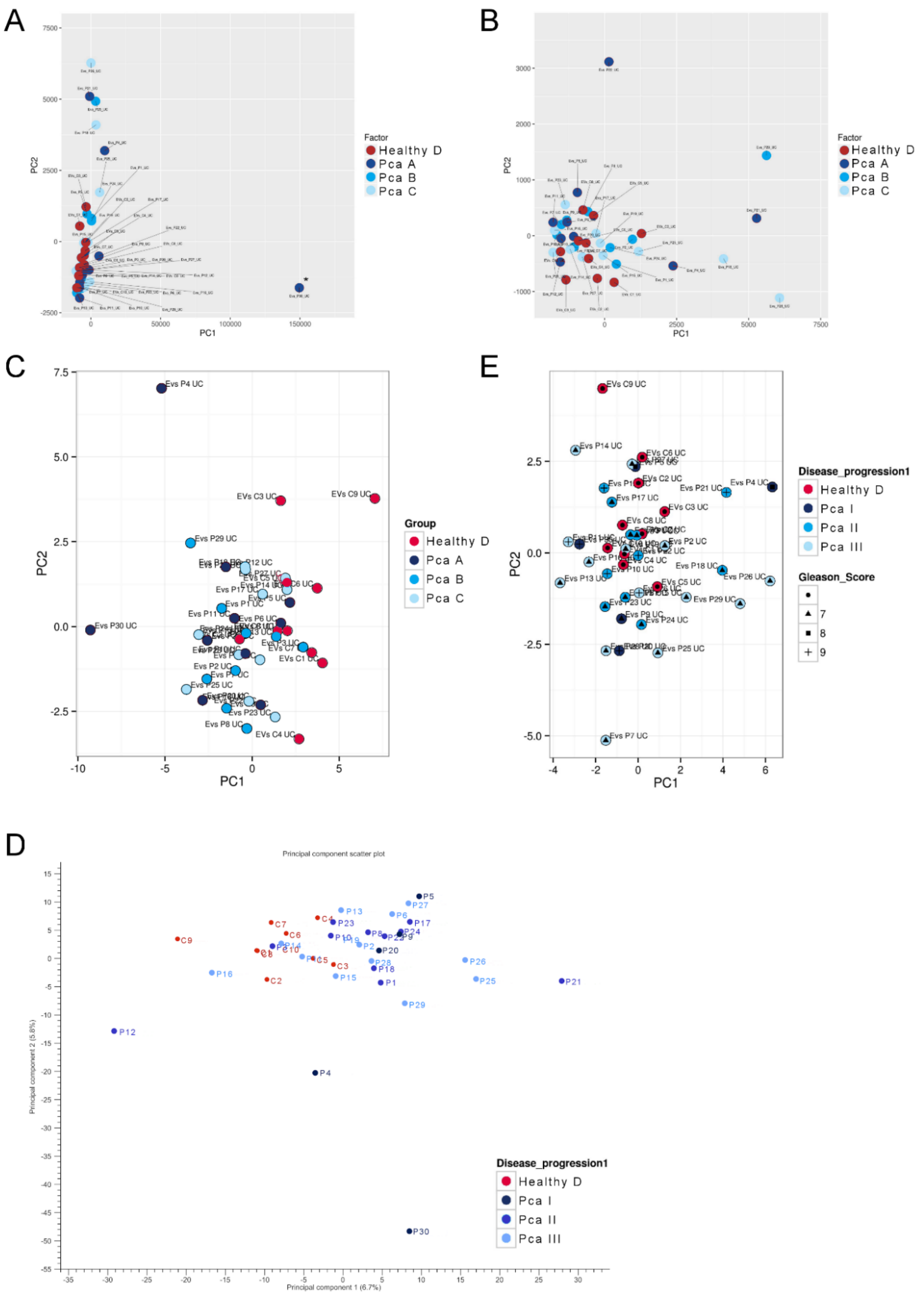

Figure S3. Principal component analysis of miRNAseq and qPCR data. Sequencing data was analyzed (**A**) with an outlier, patient 30 (P30)*, or (**B**) without based on PCa status groups A–C and healthy controls group D. (**C**) Analysis of qPCR data for candidate differential and reference miRNAs in the study of PCa status groups. (**D**) The miRNAseq data was reanalyzed based on PCa progression groups (I-III). (**E**) qPCR data for candidate differential and reference miRNAs in the study of PCa progression groups. All PC analysis were based on miRNAs with the largest variation: 50 miRNAs in (**A**,**B**,**D**), and 28 and 21 miRNAs in (**C**,**E**), respectively. P30 was included in (**C**–**E**). Control (C), extracellular vesicles (EVs), micro RNA sequencing (miRNAseq), patient (P), principal component (PC), prostate cancer (PCa), ultracentrifugation (UC), quantitative polymerase chain reaction (qPCR).

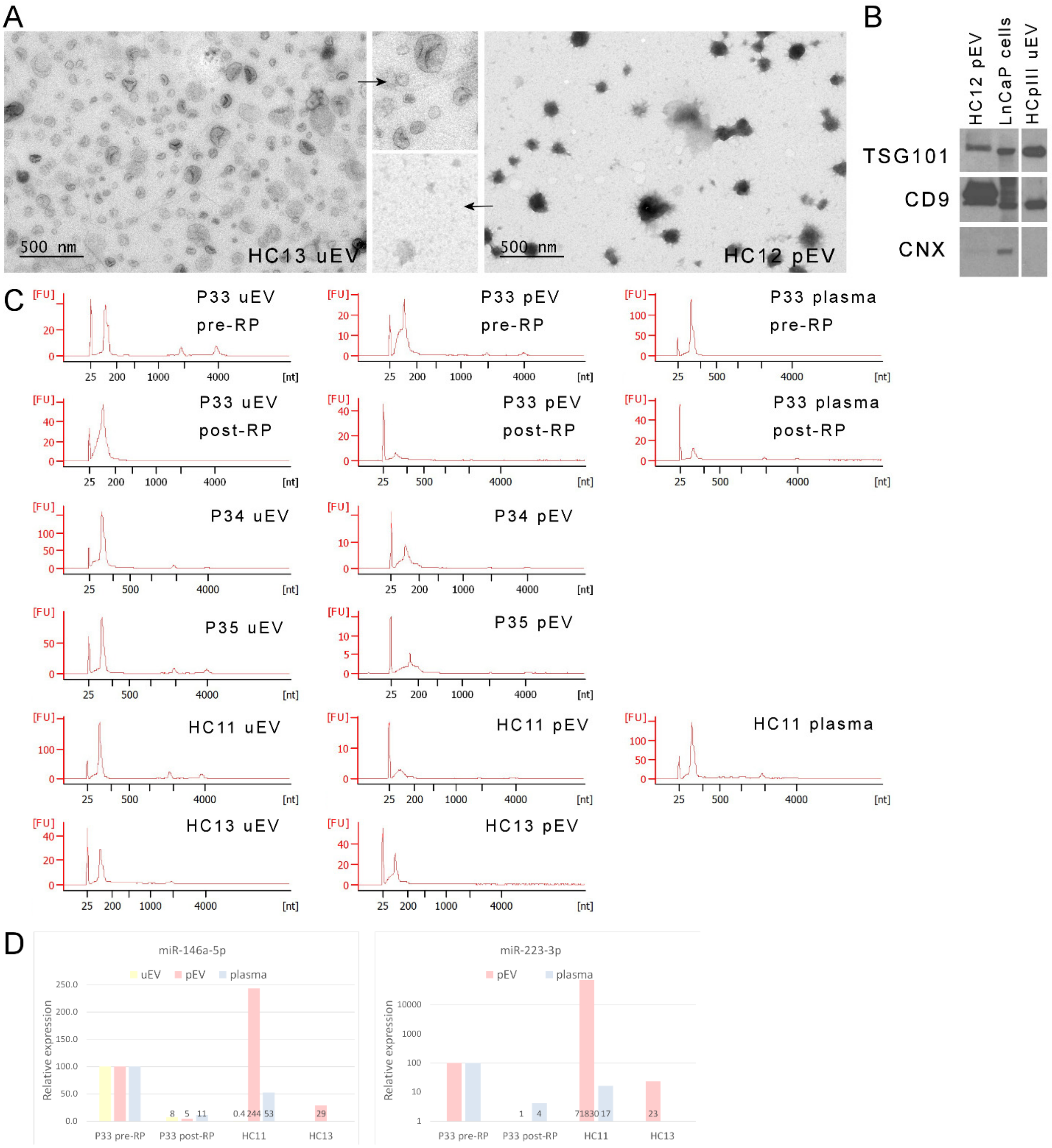

Figure S4. Quality control and miRNA expression of the correlation study samples. Representative (**A**) electron micrographs (wide-field and close-up) and (**B**) Western blotting of EV enriched proteins (CD9, TSG101) or calnexin (CNX) in control uEV and pEV samples and LnCaP cells. (**C**) Total RNA profiles from all correlation study samples obtained by Bioanalyzer Pico assay. (**D**) Relative expression of miR-146a-5p and -223-3p in miRNA sequencing. Healthy control (HC), HC pool III (HCpIII), patient (P), plasma EV (pEV), before prostatectomy (pre-RP), after prostatectomy (post-RP), urine EV (uEV).

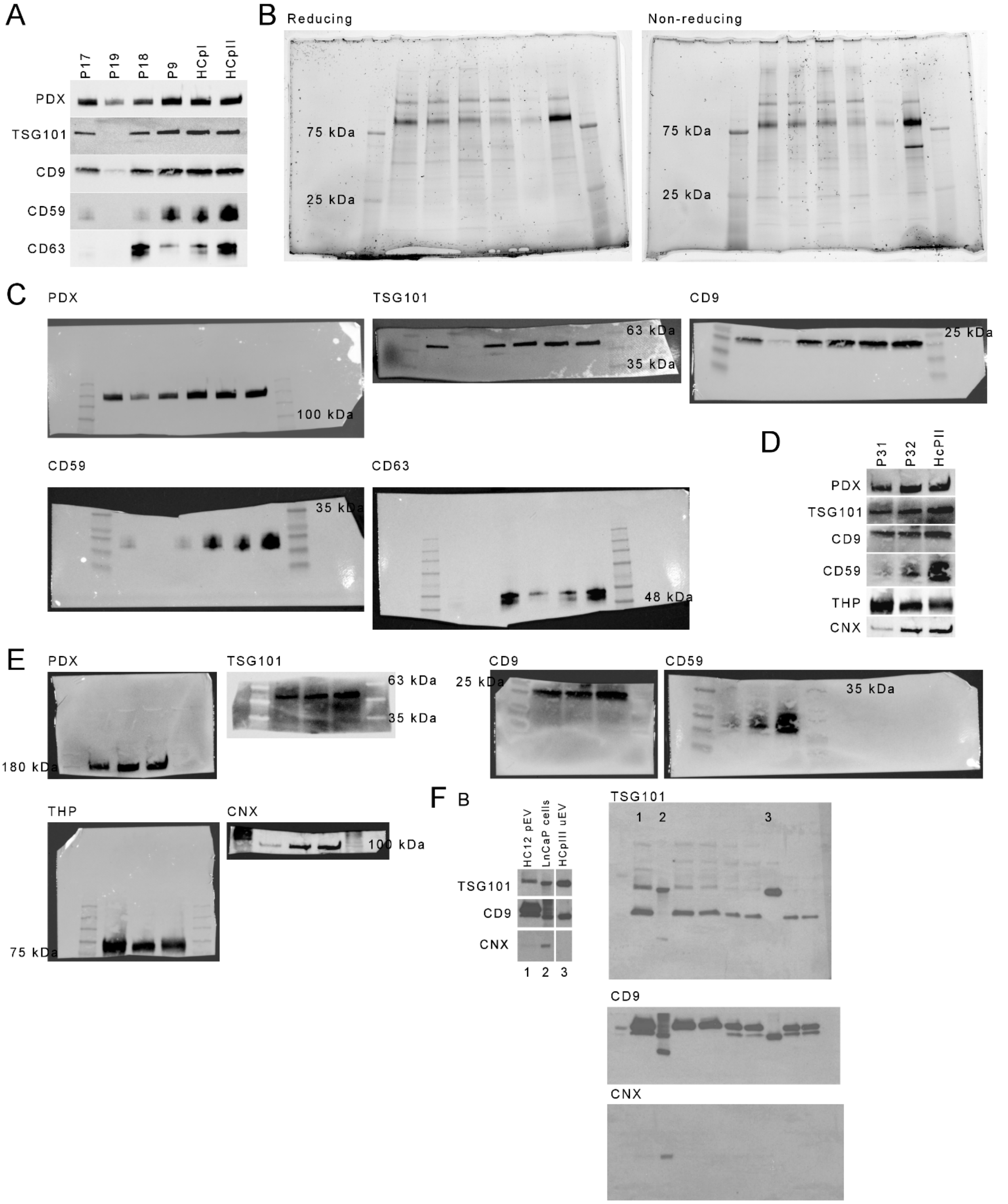

Figure S5. Western blotting originals. (**A**) Compilation of Western blotting bands of uEV samples presented in Figure 2. (**B**) Protein images of study samples (in A and Figure 2) in reducing and non-reducing sample buffer conditions in SDS-PAGE gels, and (**C**), the respective raw images from Western blotting including marker bands. (**D**) Compilation of Western blotting bands presented in Figure S1, and (**E**), the respective raw Western blotting images including marker bands. (**F**) Western blotting presented in Figure S4 and the respective raw images (numbered lanes). Sample order shown in (**A**) applies to (**B**,**C**). Sample order shown in (**D**) applies to (**E**). Molecular weight marker bands have approximate molecular weights of 245, 180, 135, 100, 75, 63, 48, 35, 25, 20, 17 and 11 kDa—bands visible in protein gels (**B**) and bands close to the blot cutting points in (**C**,**E**) are indicated.
